# Indications to perform damage control surgery in pediatric trauma: a scoping review—Are children little adults?

**DOI:** 10.1186/s13017-025-00647-x

**Published:** 2025-10-27

**Authors:** Kris R. Wiendels, Joris Lemson, Manouk Backes, Erik Hermans, Jan Bollen, Diederik P. J. Smeeing, Stijn D. Nelen

**Affiliations:** 1 Department of Surgery, Radboudumc, Nijmegen, The Netherlands; 2 Department of Intensive Care Medicine, Radboudumc, Nijmegen, The Netherlands; 3 Department of Anesthesiology, Pain and Palliative Care, Radboudumc, Nijmegen, The Netherlands; 4 Department of Surgery, Rijnstate Hospital, Arnhem, The Netherlands

**Keywords:** Pediatric, Trauma, Damage control surgery, Damage control intervention, Indication, Lethal triad, Scoping review

## Abstract

**Background:**

Damage Control Surgery is a technique aimed at reducing mortality in trauma patients, but its use in pediatric patients lacks standardized indications. Proper patient selection is essential to mitigate morbidity associated with Damage Control Surgery.

**Objective:**

This review aims to clarify the reported indications for Damage Control Surgery in pediatric trauma patients.

**Methods:**

A systematic search of PubMed and Embase was conducted without publication year restrictions to identify studies reporting indications for performing Damage Control Surgery in pediatric trauma patients. Backward citation analysis was performed on identified review articles that were excluded. Indications or patient characteristics guiding surgical decision-making in the emergency department were extracted and categorized.

**Results:**

Forty studies were included: 25 case reports, 13 case series, and 2 observational studies. The case reports and case series involved 98 patients with 368 reported indications, with severe trauma (26.1%), hemodynamic instability (18.2%), and radiological or clinical evidence of severe hemorrhage or contamination (28.2%) being the most observed. The observational studies found a higher Injury Severity Score, lower systolic blood pressure, decreased Glasgow Coma Scale, lower body temperature, and more frequent blood transfusions in the Damage Control Surgery groups compared to the control groups.

**Conclusions and relevance:**

Severe trauma, hemodynamic instability, and injuries related to severe hemorrhage or contamination emerged as key indications for Damage Control Surgery in pediatric trauma, consistent with findings in adult trauma populations. However, the lethal triad of acidosis, hypothermia and coagulopathy was infrequently reported as a primary indication for Damage Control Surgery in children. This may reflect the greater compensatory capacity of pediatric patients, potentially delaying the manifestation of these physiological derangements. Our findings suggest that early intervention with Damage Control Surgery in cases of severe trauma, exsanguination, gross contamination, and hemodynamic instability may help prevent the progression to critical physiological states such as the lethal triad. This underscores the importance of timely recognition and intervention in pediatric trauma management.

**Supplementary Information:**

The online version contains supplementary material available at 10.1186/s13017-025-00647-x.

## Background

**Fig. 1:**
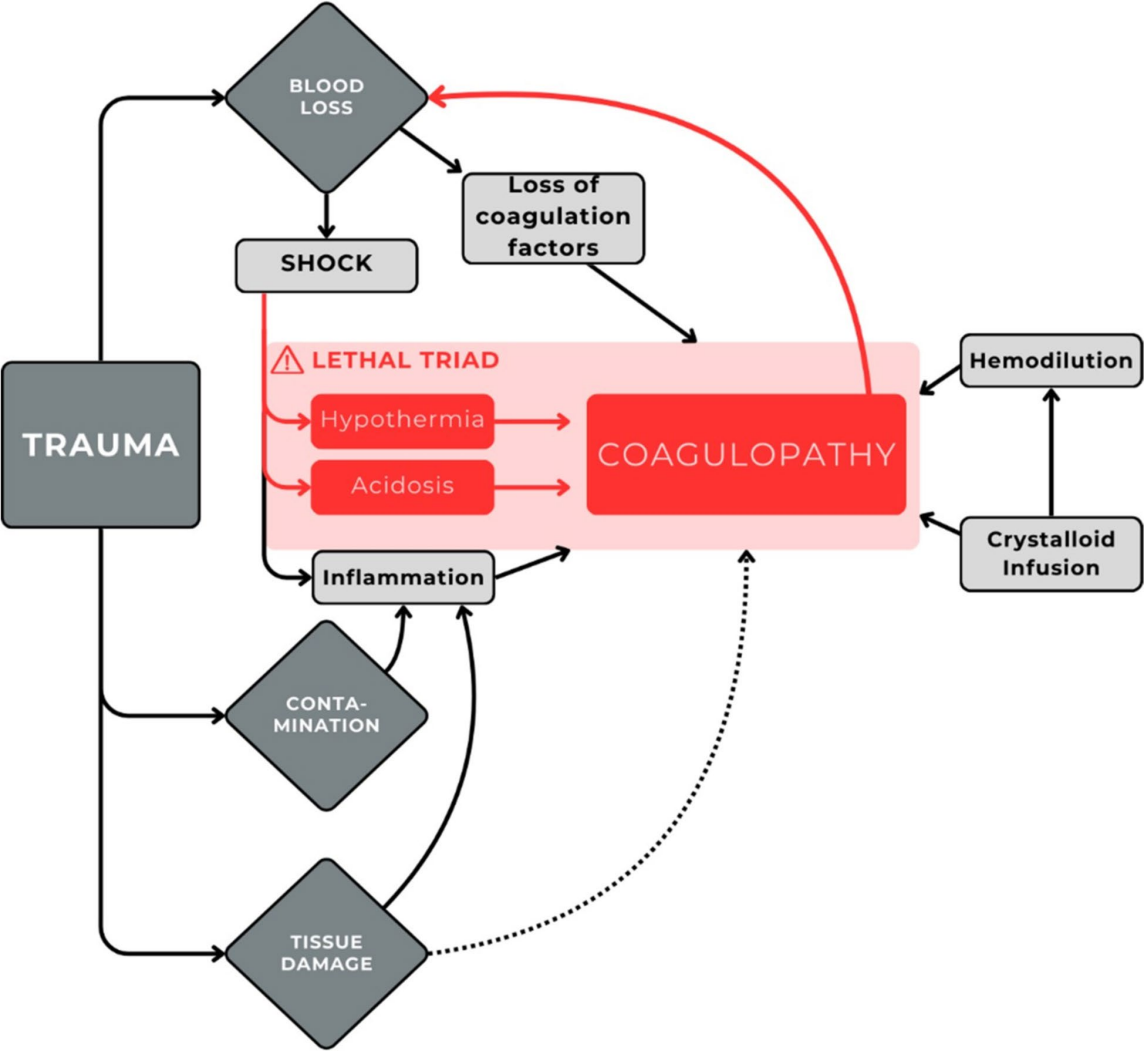
Pathophysiology of trauma. Trauma leads to both significant blood loss and an inflammatory response, resulting in shock with hypotension and poor tissue perfusion. This progresses to the lethal triad of coagulopathy, hypothermia, and acidosis, with the latter two exacerbating coagulopathy. Various iatrogenic factors can worsen this process (e.g., cold fluids, cold environment, transfusion ratios, sedation). Coagulopathy represents the final endpoint, leading to a vicious cycle with increased hemorrhage and worsening of coagulopathy

Damage Control Surgery (DCS) is defined as staged surgery to control hemorrhage and contamination, enabling resuscitation in the Intensive Care Unit (ICU) with subsequent definitive surgical repair once the patient’s physiology is stabilized [1–3]. This strategy was developed to counteract the lethal triad of acidosis, hypothermia, and coagulopathy, which are strongly associated with mortality in severely injured patients (Fig. 1) [3–6].

Although DCS has demonstrated a decrease in mortality in recent decades, it remains associated with significant morbidity, necessitating careful consideration of its use as a treatment option [2, 7–10]. This presents a challenge in the emergency department (ED), where the time-sensitive nature of care limits the opportunity for extensive decision-making. This emphasizes the need for clear and effective guidelines in (pediatric) trauma care.

Roberts et al. have extensively studied the indications for DCS, identifying several preoperative criteria such as hypotension, hypothermia, acidosis, transfusion requirements, high Injury Severity Score (ISS), and penetrating trauma to the upper abdomen [11–14]. However, specific pediatric indications are underrepresented in these studies. Given the anatomical and physiological differences between children and adults, as well as distinct factors influencing outcomes, pediatric indications for DCS may not align with those in adults [15–20]. This underscores the importance of further investigating the specific indications for DCS in pediatric trauma.

The aim of this review was to provide an overview and clarification of the reported indications in the ED for performing DCS in pediatric trauma patients, with the goal of suggesting a practical guideline outlining indications for DCS in children.

## Methods

### Study design

A scoping review was conducted to address the existing knowledge gap and include a broader array of study designs compared to a systematic review. This approach enabled the collection of more diverse data [21]. The review was reported in accordance with the framework by Arksey and O’Malley and followed the Preferred Reporting Items for Systematic reviews and Meta-Analyses extension for Scoping Reviews (PRISMA-ScR) checklist [21, 22].

### Eligibility criteria

Eligible studies included observational research, case reports, and case series describing the use of DCS or related interventions for traumatic injuries in patients under 18 years of age. Inclusion required that the full text specified indications or patient characteristics present in the ED that influenced surgical decision-making.

An operative intervention was classified as DCS if the authors explicitly described it as such. If not explicitly stated, the procedure was considered DCS when all the following criteria were met:

- Emergency surgery within 24 h of the injury
- A staged surgical approach with re-exploration during the same hospital admission
- The initial procedure aimed at controlling hemorrhage and/or contamination

Controlling contamination was defined as staged surgery for Gustilo–Anderson (GA) grade III fractures with extensive soft tissue damage, hollow viscus injury, or contaminated penetrating thoraco-abdominal wounds. Grade III open fractures were selected as a criterion because of their association with high-energy trauma, concomitant vascular and nerve injuries, and substantial contamination.

A distinction was made between Damage Control Orthopedics (DCO), focusing on fracture management, and Damage Control Laparotomy (DCL) or Thoracotomy (DCT), which addressed intra-abdominal or thoracic injuries.

Exclusion criteria included non-traumatic indications for DCS (e.g., thermal injury, abdominal sepsis), staged procedures to treat traumatic wounds without fractures or internal organ damage (e.g., fasciotomy, degloving injuries), and surgery performed for reasons other than controlling hemorrhage or contamination (e.g., neurosurgery, acute spine surgery). Systematic reviews and meta-analyses were excluded from direct inclusion but were used for backward citation analysis.

### Information sources

A systematic search in PubMed/MEDLINE and Embase was conducted for articles published up to April 17th, 2025. There were no publication date restrictions; only studies published in English or Dutch were included. The search strategy was developed in collaboration with an information specialist and is detailed in Appendix A.

### Selection of sources of evidence

Two reviewers (KW and DS) independently screened titles and abstracts. Articles were included if there was a reasonable indication that DCS or Damage Control interventions (e.g., external fixation as a bridge to definitive fixation, temporary abdominal closure, abdominal packing, temporary arterial shunts) were used to manage pediatric trauma. These two reviewers then independently assessed whether the full text met the eligibility criteria. Disagreements were resolved through discussion or, if needed, by a senior author (SN). The selection process was conducted using Rayyan’s systematic review tool [23].

### Data charting process

A structured codebook was developed to guide data extraction (Appendix B). Indications and patient characteristics were coded into predefined categories based on the framework by Roberts et al. [12], including injury pattern, degree of physiological insult, and ED-based resuscitation or interventions. The injury pattern included clinical findings, imaging results, and anatomical trauma scores. The degree of physiological insult referred to abnormalities in vital signs, physical examination, and laboratory values. A residual category captured indications not fitting these groups. Details of the extraction and coding process are provided in Appendix C.

### Data items

Extracted data included author(s), publication year, country, study design, intervention type (e.g., DCL or DCO), comparator (e.g., definitive surgery), patient characteristics, and outcomes of interest. In the case reports and case series, outcomes were the reported indications for DCS or, if this was not explicitly stated, the patient characteristics which were likely involved in surgical decision making. In the observational studies, outcomes were statistically significant differences in patient characteristics between the DCS and definitive surgery groups. Only indications or patient characteristics that were present in the ED were included.

### Critical appraisal

Study quality was graded using the Oxford 2011 Levels of Evidence [24]. The Methodological Index for Non-Randomized Studies (MINORS) was performed to assess the observational studies [25].

### Synthesis of results

Study and patient characteristics were summarized using frequencies and proportions. Age distribution was described using mean, standard deviation (SD), median, and interquartile range (IQR). Reported indications in the case reports and case series were presented in frequencies and percentages. The categorization of indications followed the structure outlined in the codebook (Appendix B). Based on the results, this structure was adjusted to reduce complexity and enhance readability.

For observational studies, statistically significant differences were extracted directly from the original articles and summarized, including the effect measures and *p*-values. Units of measurement were standardized. All outcomes were categorized according to the framework by Roberts et al. [12]. All analyses were performed using IBM SPSS Statistics 27.

## Results

### Selection process

**Fig. 2:**
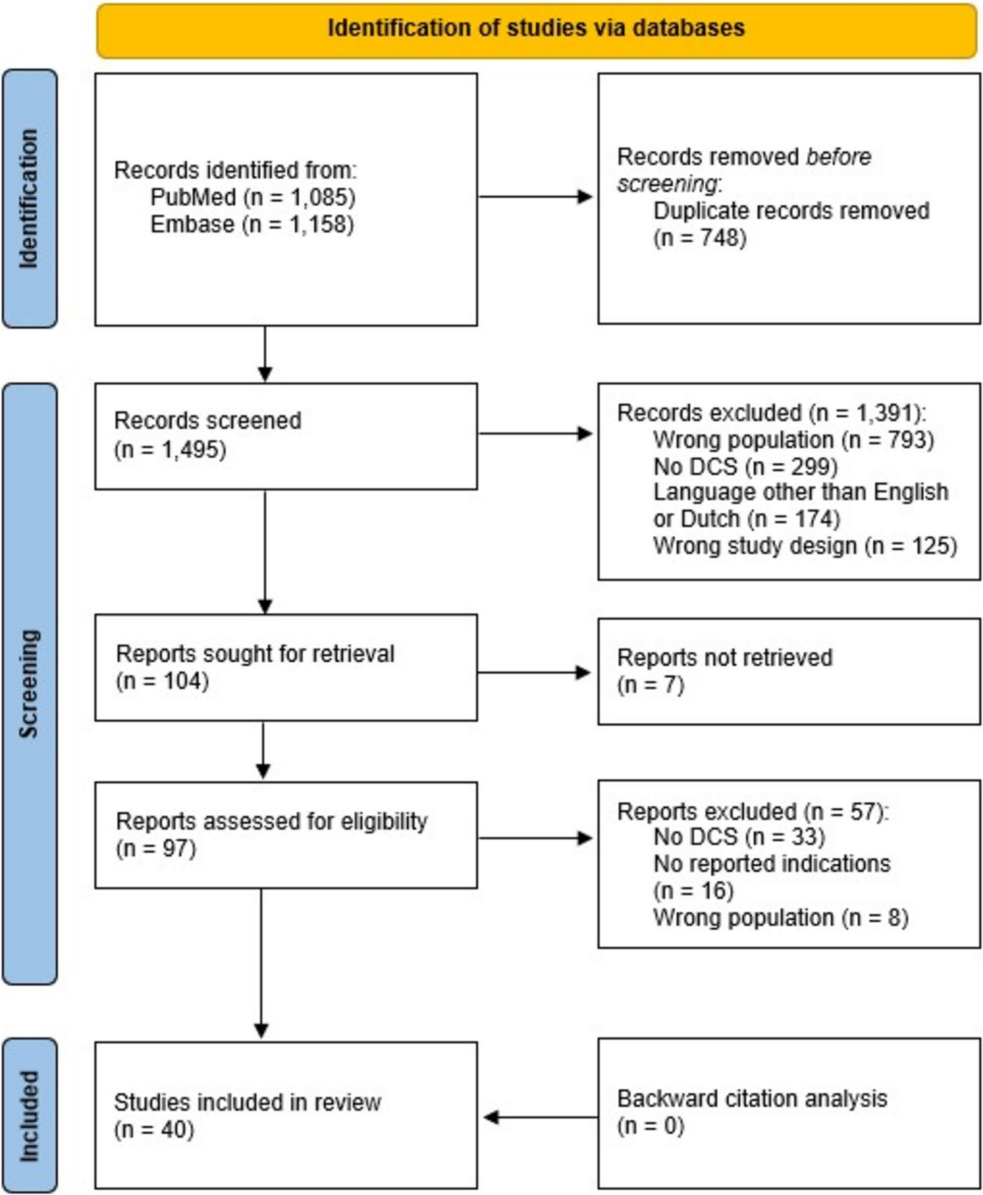
Flowchart of study selection

Of the 2243 studies identified, 1495 were screened after removing duplicates. Subsequently, 97 full-text articles were assessed for eligibility. Backward citation analysis did not yield any additional studies. In total, 40 studies were included (Fig. 2).

### Study and patient characteristics

The 40 included studies consisted of 25 case reports, 13 case series, and 2 observational studies, meaning that the majority represented level 4 or 5 evidence. The quality of the observational studies was assessed using the MINORS tool, as detailed in Appendix D.

Most studies originated from the United States of America (n = 21), were published after 2010 (n = 31), and focused on DCL (n = 25). The case reports and case series included a total of 98 eligible patients, with 368 reported indications. Age and sex were reported for 83 patients, and the mechanism of injury for 61 patients. The mean age was 9.9 years, and 63.9% were male. The most common mechanisms of injury were road traffic accidents (45.9%), falls from height (24.6%), and gunshot wounds (14.8%).

Both observational studies compared DCL with direct definitive surgery and included a total of 416 patients. These studies showed a different age distribution compared to the case reports and case series, with both median and mean ages around 16 years. The average proportion of male patients was 76.0%.

A summary of the study and patient characteristics is presented in Table 1. A comprehensive overview of all included studies, their individual characteristics, and the extracted data is available in Appendix C.

**Table 1:** Overview of study and patient characteristics

Characteristic	Value	
**Study characteristics (n = 40)**		
Study type and quality of evidence, n (%)		
Observational study (Level 3)	2	5.0
Case series (Level 4)	13	32.5
Case report (Level 5)	25	62.5
Year of publication, n (%)		
≥ 2020	21	52.5
2010–2019	10	25.0
2000–2009	5	12.5
≤ 1999	4	10.0
Country of origin, n (%)		
USA	21	52.5
China	4	10.0
Japan	4	10.0
Other (all n = 1)	11	27.5
Primary intervention type, n (%)		
DCL	26	65.0
DCO	14	35.0
**Patient characteristics case reports and case series (n = 98)**		
Age in years, mean (SD)	9.9	4.4
Sex, n (%)^a^		
Male	53	63.9
Female	30	36.1
Mechanism of injury, n (%)^b^		
Road traffic accident	28	45.9
Fall from height	15	24.6
Gunshot wound(s)	9	14.8
Other	9	14.8
**Patient characteristics observational studies (n = 416)**^c^		
Age in years		
Polites et al. mean (SD)	16	4
Villalobos et al. median (IQR)	16.5	14–18
Sex, n (%)		
Male	316	76.0
Female	100	24.0

DCL, damage control laparotomy; DCO, damage control orthopedics; DCS, damage control surgery; IQR, interquartile range; SD, standard deviation; USA; United States of America

^a^The sex category was only presented for 83 out of 98 patients

^b^The mechanism of injury was only presented for 61 out of 98 patients

^c^The patient characteristics are presented for the patients who underwent DCS. The mechanism of injury was not traceable from the observational studies

### Reported indications in the case reports and case series

A total of 368 indications were extracted from the included case reports and case series. Most of these were categorized under either injury pattern (n = 202, 54.9%) or the degree of physiological insult (n = 132, 35.9%). The most frequently reported indications included GA grade III fractures requiring staged surgical management (n = 55, 14.9%), multitrauma involving multiple long bone or axial skeleton fractures and/or internal organ injuries (n = 40, 10.9%), hypotension (n = 29, 7.9%), unstable pelvic fractures (n = 25, 6.8%), and an ISS ≥ 15 (n = 24, 6.5%). Among these, GA grade III and unstable pelvic fractures emerged as prominent indications for patients who underwent DCO.

When grouped into broader subcategories, 72.5% of the indications were related to severe trauma (n = 96, 26.1%), clinical or radiological evidence of severe hemorrhage or contamination (n = 41, 11.1%, and n = 63, 17.1%, respectively; combined 28.2%), or hemodynamic instability (n = 67, 18.2%). A detailed overview of all reported indications from the case reports and case series is presented in Table 2 [26–63].

**Table 2:** Reported indications in case reports and case series

Reported indication	n	%
Total	368	100.0
**Injury pattern**		
Severe trauma	**96**	**26.1**
Multitrauma: Multiple fractures and/or internal organ injuries‡	40	10.9
Unstable pelvic fracture‡	25	6.8
ISS ≥ 15‡	24	6.5
Penetrating truncal injury	7	1.9
Severe hemorrhage	**41**	**11.1**
Ultrasonography: Abdominal fluid collection	21	5.7
CT: Contrast extravasation or major vessel injury	7	1.9
CT: Intra- or retroperitoneal fluid collection	6	1.6
CT: Severe solid organ injury	5	1.4
Clinical evidence of severe hemorrhage	2	0.5
Severe contamination	**63**	**17.1**
GA grade III fracture(s) requiring staged surgery ‡	55	14.9
CT: Hollow-viscus injury or abdominal free air	8	2.2
Other (all n ≤ 2)	**2**	**0.5**
**Degree of physiological insult**		
Hemodynamic instability	**67**	**18.2**
Hypotension ‡	29	7.9
Poor response to fluid resuscitation	15	4.1
Tachycardia	12	3.3
Deterioration of initial hemodynamic parameters	7	1.9
Poor response to pelvic binding	3	0.8
CPR on arrival	1	0.3
Non-hemodynamic vital signs	**23**	**6.3**
GCS < 15 or altered consciousness	7	1.9
Tachypnea	5	1.4
Hypothermia	5	1.4
Other (all n ≤ 2)	6	1.6
Abnormal laboratory parameters	**27**	**7.3**
Acid–base disturbances: Low pH, high lactate or low BE	13	3.5
Anemia or drop in Hb/Ht	7	1.9
Abnormal coagulation studies	4	1.1
Other (all n ≤ 2)	3	0.8
Abnormal physical exam	**15**	**4.1**
Abdominal pain or tenderness	7	1.9
Abdominal distention	5	1.4
Other (all n ≤ 2)	3	0.8
**Resuscitation or interventions given in the ED**		
Crystalloid fluid resuscitation	11	3.0
Blood transfusion	8	2.2
Intubation	5	1.4
Other (all n ≤ 2)	9	2.5
**Other**		
PTS ≤ 8	1	0.3

Table with the reported indications in the case reports and case series. Indications mentioned ≤ 2 times are not listed separately in this table, but can be found in Appendix C. Subcategories are presented in bold, with cumulative frequencies reflecting the sum of individual indications classified within each subcategory

BE, base excess; CPR, cardiopulmonary resuscitation; CT, computer tomography; ED, emergency department; GA, Gustilo–Anderson; GCS, Glasgow coma scale; ISS, injury severity score, PTS; pediatric trauma score

‡ Top 5 reported indications

### Reported indications in observational studies

In the observational studies, fifteen distinct patient characteristics were found to differ significantly between patients undergoing DCL and those receiving direct definitive surgery. Of these, eleven (73.3%) overlapped with the indications identified in the case reports and case series. Characteristics not reported in the case studies included thoracic injury, extremity trauma, traumatic brain injury, and the Penetrating Trauma Index (PATI).

Most of the patient characteristics fell under the categories of injury pattern or degree of physiological insult. Both studies reported that patients in the DCL group had a higher ISS, lower systolic blood pressure, decreased Glasgow Coma Scale (GCS), lower body temperature, and higher frequency of blood transfusions. The results of the observational studies are summarized in Table 3 [64, 65].

**Table 3:** Reported indications in observational studies

Patient characteristic	Polites et al.			Villalobos et al.		
	DCL	DDS	p	DCL	DDS	p
Injury pattern						
ISS ‡	25**	18**	< 0.001	33**	16**	< 0.001
Major vessel injury, %	NA	NA	NA	35.7	5.7	< 0.001
Pelvic fracture, %	NA	NA	NA	35.7	13.3	< 0.001
Thoracic injury, %	NA	NA	NA	67.9	32.1	< 0.001
TBI, %	NA	NA	NA	30.4	24.8	0.010
Solid organ injury, %	NA	NA	NA	62.5	46.7	0.031
Extremity trauma, %	NA	NA	NA	37.5	24.4	0.049
Degree of physiological insult						
SBP, mmHg ‡	104*	113*	< 0.001	93**	122**	< 0.001
GCS ‡	12*	13*	< 0.001	11.5**	15**	< 0.001
Temperature, degrees Celsius ‡	34*	36*	< 0.001	36.2**	36.7**	0.007
Heart rate, bpm	112*	100*	< 0.001	NA	NA	NA
BE, mmol/L	NA	NA	NA	− 8**	− 5**	< 0.001
Resuscitation or interventions given in the ED						
Blood transfusion, % ‡	19	11	< 0.001	44.6	9.5	< 0.001
Crystalloid fluid infusion, mL	NA	NA	NA	2000**	700**	< 0.001
Other						
PATI score	NA	NA	NA	29	8	< 0.001

The reported indications represent significant differences (*p* < 0.05) in patient characteristics between the DCL and DDS groups. Polites et al. included 360 patients in the DCL group and 2174 in the DDS group; Villalobos et al. included 56 and 315 patients in the DCL and DDS groups, respectively

The critical appraisal scores were 9/16 for Polites et al. and 12/16 for Villalobos et al. using the MINORS tool (Appendix D)

BE, base excess; DCL, damage control laparotomy; DDS, direct definitive surgery; ED, emergency department; GCS, Glasgow coma sca; ISS, injury severity score, NA, not applicable; PATI, penetrating trauma index; SBP, systolic blood pressure; TBI, traumatic brain injury

*Mean, **Median, ‡Characteristics that were significant in both studies

## Discussion

### Key results

The aim of this review was to provide an overview of the reported indications for performing Damage Control Surgery (DCS) in pediatric trauma patients. The findings indicate that key factors influencing surgical decision-making include:

1. *Severity of traumatic lesions*, such as an Injury Severity Score (ISS) ≥ 15 (or Abbreviated Injury Score [AIS] ≥ 4), multiple fractures or internal organ injuries, unstable pelvic fractures, or penetrating truncal injuries. While ISS and AIS are widely used in research to quantify trauma severity [66], they rely on complete injury assessment and are not available during early clinical decision-making. In practice, surgeons base their judgment on trauma mechanism and presentation. We recommend using indicators such as suspected high-energy impact (e.g., high-speed collisions), multi-system trauma (e.g., extensive blunt injury), or severe localized injury (e.g., penetrating wounds) as practical markers of severe trauma.
2. *Clinical or radiological evidence of exsanguination or contamination*, including major vascular injury, abdominal free fluid, hollow viscus injury, or Gustilo–Anderson grade III fractures requiring staged management.
3. *Hemodynamic instability*, characterized by hypotension, tachycardia, indicators of poor tissue perfusion or insufficient response to resuscitation.

Both observational studies reported differences in Glasgow Coma Scale (GCS). However, the difference reported by Polites et al. was not deemed clinically relevant due to its small magnitude [64].

### Pediatric versus adult indications

Compared to the predominantly adult trauma population described by Roberts et al., most indications similarly fall into the categories of injury pattern and degree of physiological insult. Consistent with our findings, hemodynamic instability and severe trauma mechanisms were reported as indications for DCS. However, Roberts et al. did not specify radiological indications, and their study had access to a larger volume of higher-quality data, allowing for more refined indications than is currently possible in pediatric trauma care [11–14].

A notable finding was the limited role of the lethal triad (coagulopathy, acidosis, and hypothermia) in our pediatric study, despite its well-established relevance in adult trauma literature [1, 3, 4, 11–14]. In the case reports and case series, these physiological factors were cited in only 22 of 368 indications (6.0%), whereas hemodynamic instability (18.2%), severe trauma (26.1%), and evidence of severe hemorrhage or contamination (28.2%) were more frequently reported. A lower body temperature was mentioned in both observational studies, although the median temperature of 36.2 °C in Villalobos et al. did not meet the threshold for hypothermia [65]. Base excess was referenced in one observational study, and coagulopathy in none [64, 65].

Table 4 compares our findings with those from the general trauma population, which primarily consists of adults. For this comparison, we refer to the content analysis by Roberts et al. [11], which builds on their earlier scoping review [12], and shares conceptual overlap with our study. It is important to note that, due to differences in methodology and coding, the results are not directly comparable. Nonetheless, they offer a meaningful approximation of the differences observed.

**Table 4:** Adult versus pediatric indications

Reported indication	Adult indications^a^		Pediatric indications		
	Roberts et al.		Case studies		Obs. studies
	N	%	N	%	N^b^
Total	141	100.0	368	100.0	2
Injury pattern					
Multiple injuries	20	14.2	40	10.9	0
High ISS	14	9.9	24	6.5	2
Pelvic fracture^c^	7	5.0	25	6.8	1
Degree of physiological insult					
Hemodynamic instability^d^	29	20.6	67	18.2	2
Lethal triad	38	27.0	22	6.0	NA
Hypothermia	14	9.9	5	1.4	1^e^
Coagulopathy	10	7.1	4	1.1	0
Acidosis	9	6.4	13	3.5	1
≥ 2 items of the lethal triad	5	3.5	NA	NA	NA
Resuscitation or interventions given in the ED					
ED thoracotomy	7	5.0	1	0.3	0
Other					
Mass casualty incident	12	8.5	NA	NA	NA

Results of the content analysis by Roberts et al. [11] are presented. All indications accounting for more than 5% (n = 7) of those reported during primary and secondary survey (n = 141) were included and subsequently compared with the results of our study. Prehospital indications were excluded. The most striking finding is the reported difference in the lethal triad

ED, emergency department; DCL, damage control laparotomy; ISS, injury severity score; NA, not applicable

^a^The study by Roberts et al. reviewed the general trauma population, yielding only limited pediatric-specific indications [12]

^b^Number of included observational studies reporting this patient characteristic as significantly different between the DCL and definitive surgery groups

^c^In Roberts et al. this refers to Tile B and C pelvic fractures; in the pediatric studies, only unstable pelvic fractures were included

^d^In Roberts et al. hemodynamic instability is primarily defined by hypotension, whereas in children the concept is broader due to physiological differences (Table 2)

^e^The study by Villalobos et al. was excluded, as median temperature in the DCL group remained within normal range

### Role of the lethal triad

Our findings suggest that the lethal triad may play a less prominent role in early decision-making for pediatric DCS than in adult cases. Among its components, coagulopathy is considered the most critical component, as it contributes to ongoing blood loss. It is driven by factors such as hemorrhage, tissue damage, shock, and inflammation [1, 67–69], which are all present in trauma. In contrast, acidosis and hypothermia may have limited impact when occurring in isolation. Acidosis in non-traumatic conditions (e.g., diabetic ketoacidosis or chronic obstructive pulmonary disease) has minimal effect on coagulation [70, 71], and its negative impact in trauma is likely mediated through concurrent tissue injury and inflammation [72]. The role of hypothermia remains ambiguous: while in vitro studies show minimal effect [73], animal studies suggest impaired hemostasis during hibernation [74]. These insights imply that acidosis and hypothermia primarily act as amplifiers of coagulopathy rather than as independent drivers. Surprisingly, coagulopathy was among the least reported indications (1.1% in case studies; absent in observational studies), suggesting that other pathophysiological triggers may play a more prominent role in initiating DCS in pediatric patients.

### Pathophysiologic considerations in pediatric trauma

In pediatric trauma, the presentation and timing of shock and the lethal triad may differ due to children’s enhanced compensatory capacity [15–17]. It is hypothesized that this greater hemodynamic resilience may delay the onset of shock and associated physiological deterioration, causing these signs to appear later than in adults. Furthermore, the inflammatory response to shock may be less pronounced and slower to develop in pediatric patients [75]. Although children may initially maintain hemodynamic stability during blood loss, their relatively lower blood volume can lead to rapid and severe decompensation, underscoring the need for timely intervention.

In DCS, the primary objective is to control hemorrhage and contamination [3–6]. Within the context of trauma pathophysiology (Fig. 1), this approach plays a crucial role in halting progression of the lethal triad [1–3]. Therefore, in cases of severe trauma, hemodynamic instability, or clear evidence of significant hemorrhage or contamination, early consideration of DCS is warranted, even if the signs of the lethal triad have not yet manifested.

### Practical guideline for the use of damage control surgery in pediatric trauma patients

**Fig. 3:**
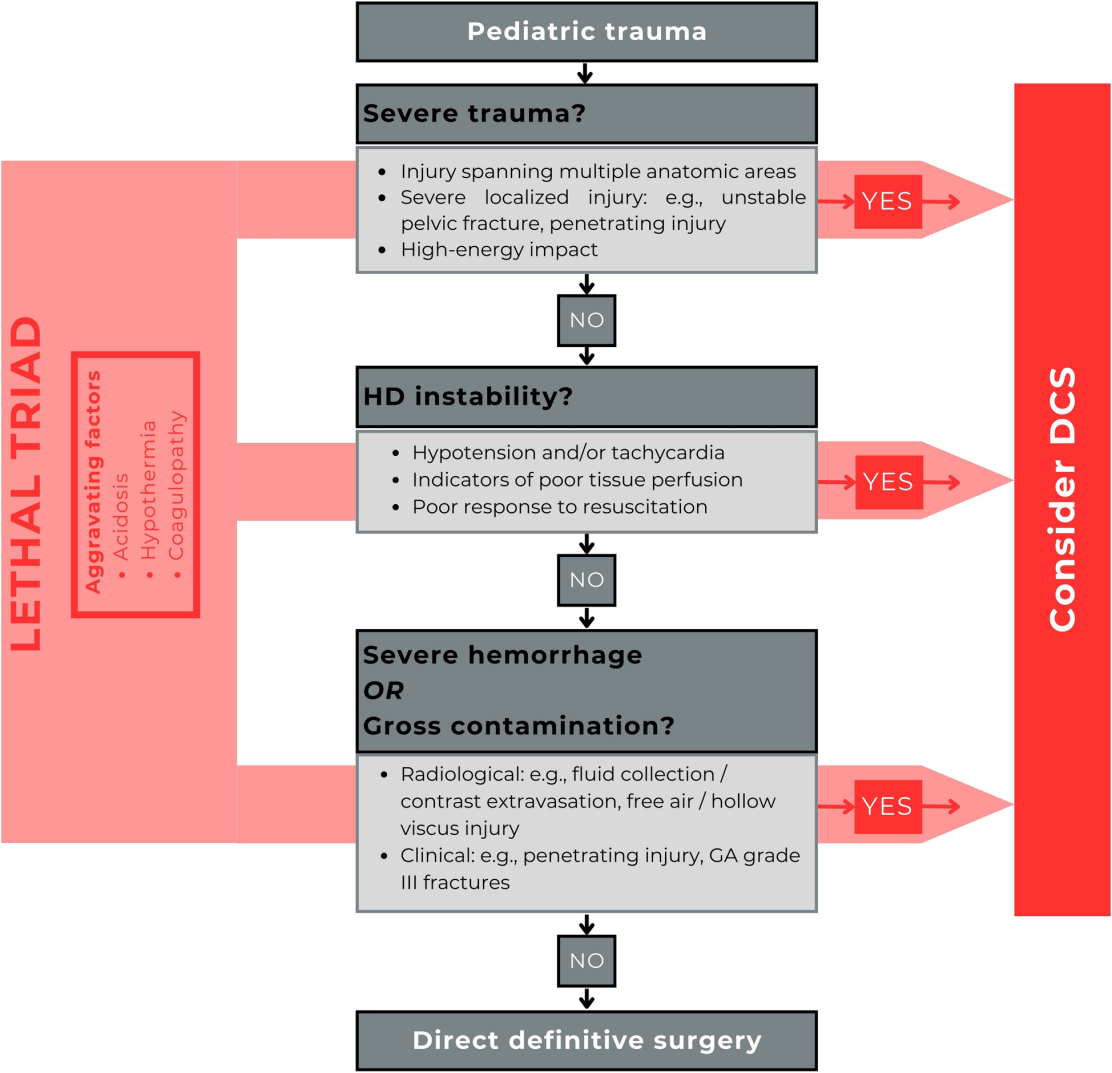
Recommendation for a practical guideline. This figure is based on the results, literature regarding the pathophysiology of trauma, and expert opinion from pediatric and trauma surgeons, pediatric intensivists, and pediatric anesthesiologists. DCS should be considered in the presence of severe trauma, hemodynamic instability, or clinical or radiological evidence of severe hemorrhage or contamination. Waiting for the presence of physiological parameters of the lethal triad is discouraged; however, if present, these factors serve as aggravating factors and should prompt immediate action. Direct definitive surgery can be justified when all the aforementioned factors are absent. Because of the risk of significant morbidity, it is important to keep reassessing whether the Damage Control approach remains justified as the patient’s clinical condition evolves. Abbreviations: AIS, Abbreviated Injury Scale; DCS, Damage Control Surgery; GA, Gustilo–Anderson; HD, Hemodynamic; ISS, Injury Severity Score

Figure 3 presents a practical guideline for determining indications for DCS in pediatric trauma patients, integrating the findings of this review, trauma pathophysiology literature, and local expert consensus. DCS should be considered in cases of severe trauma, hemodynamic instability, or clear signs of exsanguination or contamination. In the absence of these factors, definitive surgery may be appropriate.

Delaying intervention until overt signs of the lethal triad appear is discouraged, as these represent advanced physiological deterioration. Instead, their presence should prompt immediate action and reinforce the indication for DCS. Early surgical intervention is essential to control hemorrhage, address contamination, and prevent further progression of the lethal triad. This approach highlights the importance of timely clinical decision-making in pediatric trauma care to improve patient outcomes.

### Strengths and limitations

This study has several strengths. A systematic methodology was applied for study selection and data extraction, and the process was transparently documented in the supplementary appendices.

However, several limitations must be acknowledged. Only two observational studies were included, resulting in a predominance of case reports and case series. This may introduce selection bias, as the included cases may not represent the full spectrum of pediatric trauma, and reporting bias, as dramatic or successful cases are more likely to be published. Moreover, case reports often provide more detailed clinical information than case series or observational studies.

Due to methodological differences, data from case reports and series could not be directly compared with the statistical data from observational studies, limiting the analysis to descriptive statistics. Additionally, the age distribution in the observational studies was skewed, with both median and mean ages around 16 years, indicating underrepresentation of younger children. This limits the generalizability of the findings, as the physiology of the youngest patients differs most significantly from that of adults [15–17].

This review focused on identifying reported indications for initiating DCS in the ED setting. It does not assess patient outcomes or compare the safety of DCS with definitive surgical management. The decision to proceed with a staged versus definitive repair remains highly individualized and depends on intraoperative findings, patient physiology, and institutional context. In adult trauma care, concerns have been raised about potential overuse of DCL due to its high complication rate. As a result, there is a growing preference for definitive management when feasible [8–10]. Therefore, ongoing reassessment throughout the DCS process is essential to ensure that the approach remains justified. In pediatric trauma care, more outcome data are needed to guide safe and effective use of DCS, and to clarify when a stepwise approach is truly warranted.

## Conclusion

This study identified severe trauma, hemodynamic instability, and evidence of exsanguination or significant contamination as key factors influencing the decision to perform Damage Control Surgery in pediatric trauma patients. These findings are consistent with patterns observed in adult trauma populations. Although the lethal triad appeared to play a less prominent role in early decision-making, timely surgical intervention remains crucial to prevent progression to critical physiological states.

To advance clinical decision-making, future research should prioritize prospective studies involving younger pediatric patients, including direct comparisons between DCS and definitive surgical management, to evaluate outcomes such as morbidity, mortality, and long-term recovery. Additionally, consensus-building methods, such as the Delphi technique, may help establish and validate clear indications for pediatric DCS, moving the field beyond expert opinion and case studies towards a stronger evidence-based practice.

## Supplementary Information

Additional file1

## Data Availability

A supplementary file is published with this study. To enhance reproducibility the search syntax, codebook and coding process is provided in Appendix A, B and C, respectively. The SPSS datafiles used and/or analyzed during the current study are available from the corresponding author on reasonable request.

## Abbreviations

BE: Base excess
COPD: Chronic obstructive pulmonary disease
CPR: Cardiopulmonary resuscitation
CT: Computer tomography
DCL: Damage control laparotomy
DCO: Damage control orthopedics
DCS: Damage control surgery
DCT: Damage control thoracotomy
DDS: Direct definitive surgery
ED: Emergency department
GA: Gustilo–Anderson
GCS: Glasgow coma scale
ICU: Intensive care unit
IQR: Interquartile range
ISS: Injury severity score
MINORS: Methodological index for non-randomized studies
NA: Not applicable
PATI: Penetrating abdominal trauma index
PRISMA-ScR: Preferred reporting items for systematic reviews and meta-analyses extension for scoping reviews
PTS: Pediatric trauma score
SBP: Systolic blood pressure
SD: Standard deviation
TBI: Traumatic brain injury
USA: United States of America
