# Supplementary material for: Indications to perform damage control surgery in pediatric trauma: a scoping review—Are children little adults?
Source: World J Emerg Surg. 2025 Oct 27;20:81. doi: 10.1186/s13017-025-00647-x (PMC12557930; doi:10.1186/s13017-025-00647-x)
Supplement: Supplementary file 1 — Additional file1 [file 13017_2025_647_MOESM1_ESM.pdf]

Supplementary

Indications to Perform Damage Control Surgery in Pediatric Trauma: A Scoping Review

Are children little adults?

Kris R. Wiendels[1], Joris Lemson, MD, PhD[2], Manouk Backes, MD, PhD[1], Erik Hermans, MD, PhD[1], Jan Bollen, LLM MD PhD[3], Diederik P.J. Smeeing, MD, PhD[4] *and* **Stijn D. Nelen, MD, PhD[1]**

[1] Department of surgery, Radboudumc, Nijmegen, The Netherlands

[2] Department of intensive care medicine, Radboudumc, Nijmegen, The Netherlands

[3] Department of anesthesiology, pain and palliative care, Radboudumc, Nijmegen, The Netherlands

[4] Department of surgery, Rijnstate hospital, Arnhem, The Netherlands

Table of contents

**APPENDIX A**: Search syntax	Pag. 2
**APPENDIX B**: Codebook for case reports and case series coding	Pag. 6
**APPENDIX C**: Overview of included studies, excluded patients and extracted data	Pag. 13
**APPENDIX D**: Critical appraisal of observational studies	Pag. 29
**REFERENCES**	Pag. 32

Submitted: 05-05-2025

Corresponding author: Kris R. Wiendels; email: kriswiendels@outlook.com

APPENDIX A: Search syntax

Key terms
Pediatric, trauma, Damage Control Surgery, mortality, morbidity, indications

Search syntax PubMed
("Pediatrics"[Mesh] OR "Infant"[Mesh] OR "Child"[Mesh] OR "Adolescent"[Mesh] OR Pediatric*[tiab] OR Infan*[tiab] OR newborn*[tiab] OR new-born*[tiab] OR baby[tiab] OR baby*[tiab] OR babies[tiab] OR toddler*[tiab] OR minor*[tiab] OR boy[tiab] OR boys[tiab] OR boyhood[tiab] OR girl*[tiab] OR kid[tiab] OR kids[tiab] OR child*[tiab] OR schoolchild*[tiab] OR school child*[tiab] OR adolescen*[tiab] OR juvenil*[tiab] OR youth*[tiab] OR teen*[tiab] OR underage*[tiab] OR pubescen*[tiab] OR paediatric*[tiab] OR peadiatric*[tiab]) NOT (Adults[Mesh])

AND

"Wounds and Injuries"[Mesh] OR Injur*[tiab] OR Trauma*[tiab] OR Wound*[tiab] OR Gunshot*[tiab] OR Stab*[tiab] OR Fracture*[tiab] OR Laceration*[tiab] OR Contusion[tiab] OR Bruise*[tiab] OR “Flail chest”[tiab] OR “stove-in chest”[tiab] OR Deglov*[tiab] OR avulsion*[tiab] OR crush[tiab]

AND

("Damage control"[tiab] OR "Damage control intervention"[tiab:~3] OR "Damage Control Interventions"[tiab:~3] OR "Damage control surgery"[tiab:~3] OR "Damage control surgeries"[tiab:~3] OR "Damage control surgeon"[tiab:~3] OR "Damage control surgeons"[tiab:~3] OR "Damage control procedure"[tiab:~3] OR "Damage control Procedures"[tiab:~3] OR "Damage control approach"[tiab:~3] OR "Damage control Approaches"[tiab:~3] OR "Damage control approaching"[tiab:~3] OR "Damage control Laparotomy"[tiab:~3] OR "Damage control Laparotomies"[tiab:~3] OR "Damage control Celiotomy"[tiab:~3] OR "Damage control Celiotomies"[tiab:~3] OR "Damage control thoracotomy"[tiab:~3] OR "Damage control Thoracotomies"[tiab:~3] OR “Damage control Orthopedics”[tiab:~3] OR “Damage control Orthopedic”[tiab:~3] OR “external fixation”[tiab:~3] OR “Damage control Urology”[tiab:~3] OR "Abbreviated surgery"[tiab:~3] OR "Abbreviated surgeries"[tiab:~3] OR "Abbreviated surgeon"[tiab:~3] OR "Abbreviated surgeons"[tiab:~3] OR "Abbreviated procedure"[tiab:~3] OR "Abbreviated Procedures"[tiab:~3] OR "Abbreviated Laparotomy"[tiab:~3] OR "Abbreviated Laparotomies"[tiab:~3] OR "Abbreviated Celiotomy"[tiab:~3] OR "Abbreviated Celiotomies"[tiab:~3] OR "Abbreviated thoracotomy"[tiab:~3] OR "Abbreviated Thoracotomies"[tiab:~3] OR "Staged surgery"[tiab:~3] OR "Staged surgeries"[tiab:~3] OR "Staged surgeon"[tiab:~3] OR "Staged surgeons"[tiab:~3] OR "Staged procedure"[tiab:~3] OR "Staged Procedures"[tiab:~3] OR "Staged approach"[tiab:~3] OR "Staged Approaches"[tiab:~3] OR "Staged approaching"[tiab:~3] OR "Staged Laparotomy"[tiab:~3] OR "Staged Laparotomies"[tiab:~3] OR "Staged Celiotomy"[tiab:~3] OR "Staged Celiotomies"[tiab:~3] OR "Staged thoracotomy"[tiab:~3] OR "Staged Thoracotomies"[tiab:~3] OR "Bailout surgery"[tiab:~3] OR "Bailout surgeries"[tiab:~3] OR "Bailout surgeon"[tiab:~3] OR "Bailout surgeons"[tiab:~3] OR "Bailout Laparotomy"[tiab:~3] OR "Bailout Laparotomies"[tiab:~3] OR "Bailout Celiotomy"[tiab:~3] OR "Bailout Celiotomies"[tiab:~3] OR "Bailout thoracotomy"[tiab:~3] OR "Bailout Thoracotomies"[tiab:~3] OR "Balloon Tamponade"[tiab:~3] OR "Balloon Catheter Tamponade"[tiab:~3] OR "temporary intravascular shunt*"[tiab] OR "intravascular shunt*"[tiab] OR "vascular shunt*"[tiab] OR "arterial shunt*"[tiab] OR "artery shunt*"[tiab] OR "temporary abdominal closure"[tiab] OR

"temporary chest closure"[tiab] OR VAC[tiab] OR "KCI VAC"[tiab] OR "vacuum assisted closure*"[tiab] OR ABThera*[tiab] OR "vacuum pack*"[tiab] OR "towel clip*"[tiab] OR "Wittmann patch"[tiab] OR "Abdomen Pack"[tiab:~5] OR "Abdominal Pack"[tiab:~5] OR "Thoracic Pack"[tiab:~5] OR "Thorax Pack"[tiab:~5] OR "Chest Pack"[tiab:~5] OR "Chests Pack"[tiab:~5] OR "Liver Pack"[tiab:~5] OR "Livers Pack"[tiab:~5] OR "Hepatic Pack"[tiab:~5] OR "Hepatics Pack"[tiab:~5] OR "Abdomen Packs"[tiab:~5] OR "Abdominal Packs"[tiab:~5] OR "Thoracic Packs"[tiab:~5] OR "Thorax Packs"[tiab:~5] OR "Chest Packs"[tiab:~5] OR "Chests Packs"[tiab:~5] OR "Liver Packs"[tiab:~5] OR "Livers Packs"[tiab:~5] OR "Hepatic Packs"[tiab:~5] OR "Hepatics Packs"[tiab:~5] OR "Abdomen Package"[tiab:~5] OR "Abdominal Package"[tiab:~5] OR "Thoracic Package"[tiab:~5] OR "Thorax Package"[tiab:~5] OR "Chest Package"[tiab:~5] OR "Chests Package"[tiab:~5] OR "Liver Package"[tiab:~5] OR "Livers Package"[tiab:~5] OR "Hepatic Package"[tiab:~5] OR "Hepatics Package"[tiab:~5] OR "Abdomen Packages"[tiab:~5] OR "Abdominal Packages"[tiab:~5] OR "Thoracic Packages"[tiab:~5] OR "Thorax Packages"[tiab:~5] OR "Chest Packages"[tiab:~5] OR "Chests Packages"[tiab:~5] OR "Liver Packages"[tiab:~5] OR "Livers Packages"[tiab:~5] OR "Hepatic Packages"[tiab:~5] OR "Hepatics Packages"[tiab:~5] OR "Abdomen Packaging"[tiab:~5] OR "Abdominal Packaging"[tiab:~5] OR "Thoracic Packaging"[tiab:~5] OR "Thorax Packaging"[tiab:~5] OR "Chest Packaging"[tiab:~5] OR "Chests Packaging"[tiab:~5] OR "Liver Packaging"[tiab:~5] OR "Livers Packaging"[tiab:~5] OR "Hepatic Packaging"[tiab:~5] OR "Hepatics Packaging"[tiab:~5] OR "Abdomen Packagings"[tiab:~5] OR "Abdominal Packagings"[tiab:~5] OR "Thoracic Packagings"[tiab:~5] OR "Thorax Packagings"[tiab:~5] OR "Chest Packagings"[tiab:~5] OR "Chests Packagings"[tiab:~5] OR "Liver Packagings"[tiab:~5] OR "Livers Packagings"[tiab:~5] OR "Hepatic Packagings"[tiab:~5] OR "Hepatics Packagings"[tiab:~5] OR "Open Abdomen"[tiab:~3] OR "Open Abdominal"[tiab:~3] OR "Open Thorax"[tiab:~3] OR "Open Thoracic"[tiab:~3] OR "Open Chest"[tiab:~3])

AND

("Mortality"[Mesh] OR "Survival"[Mesh] OR Mortalit*[tiab] OR Surviv*[tiab] OR Death*[tiab] OR End of life[tiab] OR Fatal*[tiab]) OR (“Morbidity”[Mesh] OR “Intraoperative Complications”[Mesh] OR “Postoperative Complications”[Mesh] OR “Long Term Adverse Effects”[Mesh] OR “Adverse effects”[Subheading] OR “complications”[Subheading] OR Morbidit*[tiab] OR adverse effect*[tiab] OR adverse event*[tiab] OR adverse reaction*[tiab] OR undesirable effect*[tiab] OR undesirable event*[tiab] OR undesirable reaction*[tiab] OR complicat*[tiab]) OR (Indication*[tiab])

Search string Embase

(Exp pediatrics/ OR exp child/ OR exp adolescent/ OR (pediatric* OR infan* OR newborn* OR new-born* OR baby* OR babies OR toddler* OR minor* OR boy OR boys OR boyhood OR girl* OR kid OR kids OR child* OR schoolchild* OR 'school child*' OR adolescen* OR juvenil* OR youth* OR teen* OR underage* OR pubescen* OR paediatric* OR peadiatric*).ti,ab,kf) NOT (Exp Adult/)

AND

Exp injury/ OR (injur* OR trauma* OR wound* OR gunshot* OR stab* OR fracture* OR laceration* OR contusion OR bruise* OR 'flail chest' OR 'stove-in chest' OR deglov* OR avulsion* OR crush).ti,ab,kf

AND

('Damage control'.ti,ab,kf) OR (((damage adj3 control adj3 intervention) OR (damage adj3 control adj3 interventions) OR (damage adj3 control adj3 surgery) OR (damage adj3 control adj3 surgeries) OR (damage adj3 control adj3 surgeon) OR (damage adj3 control adj3 surgeons) OR (damage adj3 control adj3 procedure) OR (damage adj3 control adj3 procedures) OR (damage adj3 control adj3 approach) OR (damage adj3 control adj3 approaches) OR (damage adj3 control adj3 approaching) OR (damage adj3 control adj3 laparotomy) OR (damage adj3 control adj3 laparotomies) OR (damage adj3 control adj3 celiotomy) OR (damage adj3 control adj3 celiotomies) OR (damage adj3 control adj3 thoracotomy) OR (damage adj3 control adj3 thoracotomies) OR (damage adj3 control adj3 orthopedic) OR (damage adj3 control adj3 orthopedics) OR (external adj3 fixation) OR (damage adj3 control adj3 urology) OR (abbreviated adj3 surgery) OR (abbreviated adj3 surgeries) OR (abbreviated adj3 surgeon) OR (abbreviated adj3 surgeons) OR (abbreviated adj3 procedure) OR (abbreviated adj3 procedures) OR (abbreviated adj3 laparotomy) OR (abbreviated adj3 laparotomies) OR (abbreviated adj3 celiotomy) OR (abbreviated adj3 celiotomies) OR (abbreviated adj3 thoracotomy) OR (abbreviated adj3 thoracotomies) OR (staged adj3 surgery) OR (staged adj3 surgeries) OR (staged adj3 surgeon) OR (staged adj3 surgeons) OR (staged adj3 procedure) OR (staged adj3 procedures) OR (staged adj3 approach) OR (staged adj3 approaches) OR (staged adj3 approaching) OR (staged adj3 laparotomy) OR (staged adj3 laparotomies) OR (staged adj3 celiotomy) OR (staged adj3 celiotomies) OR (staged adj3 thoracotomy) OR (staged adj3 thoracotomies) OR (bailout adj3 surgery) OR (bailout adj3 surgeries) OR (bailout adj3 surgeon) OR (bailout adj3 surgeons) OR (bailout adj3 laparotomy) OR (bailout adj3 laparotomies) OR (bailout adj3 celiotomy) OR (bailout adj3 celiotomies) OR (bailout adj3 thoracotomy) OR (bailout adj3 thoracotomies) OR (balloon adj3 tamponade) OR (balloon adj3 catheter adj3 tamponade)).ti,ab,kf) OR (('temporary intravascular shunt*' OR 'intravascular shunt*' OR 'vascular shunt*' OR 'arterial shunt*' OR 'artery shunt*' OR 'temporary abdominal closure' OR 'temporary chest closure' OR VAC OR 'KCl VAC' OR 'vacuum assisted closure*' OR ABThera* OR 'vacuum pack*' OR 'towel clip*' OR 'wittmann patch').ti,ab,kf) OR (((abdomen adj5 pack) OR (abdominal adj5 pack) OR (thoracic adj5 pack) OR (thorax adj5 pack) OR (chest adj5 pack) OR (chests adj5 pack) OR (liver adj5 pack) OR (livers adj5 pack) OR (hepatic adj5 pack) OR (hepatics adj5 pack) OR (abdomen adj5 packs) OR (abdominal adj5 packs) OR (thoracic adj5 packs) OR (thorax adj5 packs) OR (chest adj5 packs) OR (chests adj5 packs) OR (liver adj5 packs) OR (livers adj5 packs) OR (hepatic adj5 packs) OR (hepatics adj5 packs) OR (abdominal adj5 package) OR (abdomen adj5 package) OR (thoracic adj5 package) OR (thorax adj5 package) OR (chest adj5

package) OR (chests adj5 package) OR (liver adj5 package) OR (livers adj5 package) OR (hepatic adj5 package) OR (hepatics adj5 package) OR (abdomen adj5 packages) OR (abdominal adj5 packages) OR (thoracic adj5 packages) OR (thorax adj5 packages) OR (chest adj5 packages) OR (chests adj5 packages) OR (liver adj5 packages) OR (livers adj5 packages) OR (hepatic adj5 packages) OR (hepatics adj5 packages) OR (abdominal adj5 packaging) OR (abdomen adj5 packaging) OR (thoracic adj5 packaging) OR (thorax adj5 packaging) OR (chest adj5 packaging) OR (chests adj5 packaging) OR (liver adj5 packaging) OR (livers adj5 packaging) OR (hepatic adj5 packaging) OR (hepatics adj5 packaging) OR (abdomen adj5 packagings) OR (abdominal adj5 packagings) OR (thoracic adj5 packagings) OR (thorax adj5 packagings) OR (chest adj5 packagings) OR (chests adj5 packagings) OR (liver adj5 packagings) OR (livers adj5 packagings) OR (hepatic adj5 packagings) OR (hepatics adj5 packagings)).ti,ab,kf) OR (((open adj3 abdomen) OR (open adj3 abdominal) OR (open adj3 thorax) OR (open adj3 thoracic) OR (open adj3 chest)).ti,ab,kf)

AND

(Exp mortality/ OR exp survival/ OR (mortalit* OR Surviv* OR Death* OR ‘End-of-life’ OR Fatal*).ti,ab,kf) OR (Exp morbidity/ OR Exp complication/ OR Exp adverse event/ OR (morbidit* OR ‘adverse effect*’ OR ‘adverse event*’ OR ‘adverse reaction*’ OR ‘undesirable effect*’ OR ‘undesirable event*’ OR ‘undesirable reaction*’ OR complicat*).ti,ab,kf) OR (Exp treatment indication/ OR (indication*).ti,ab,kf)

Filter: no conference abstracts

APPENDIX B: Codebook for case reports and case series coding

The coding system was developed based on previous work by Roberts et al. (1, 2). This study formed the basis for the four main categories: injury pattern, degree of physiological insult, resuscitation/interventions given in the Emergency Department (ED), and a residual category (Other). Subcategories were defined based on both the literature and clinical rationale. Most individual indications were pre-specified (e.g., vital signs, laboratory values, basic forms of resuscitation, and general injury patterns). During data extraction, additional indications were added to the codebook if they were mentioned more than twice. Indications mentioned two times or less were retained as part of a residual group within their respective subcategory. Notes accompanying each indication specify how the respective indication was defined, unless the indication was considered self-explanatory.

To present the results in the manuscript, the indicators and subcategories were refined and reorganized based on the obtained findings, in order to reduce overall complexity and enhance readability.

**Code**	**Category**	**Subcategory (first level)**	**Subcategory (second level)**	**Indication**	**Notes**
I	Injury pattern				Clinically observable injuries, radiological findings, and anatomical trauma scores.
I_ST	Injury pattern	Severe trauma			Severe trauma defined as an ISS ≥ 15, AIS ≥ 4, multitrauma or penetrating truncal injury.
I_ST1	Injury pattern	Severe trauma		ISS ≥ 15	ISS ≥ 15 or AIS ≥ 4 (= ISS 16).
I_ST2	Injury pattern	Severe trauma		Multitrauma	Presence of significant injuries (fractures of long bones or axial skeleton excluding ribs, and/or internal organ injury) in at least two distinct anatomical regions. Fractures in contiguous bones within the same limb (e.g., tibia and fibula) are counted as a single location. Rib, hand, and foot fractures, as well as isolated soft tissue injuries, are excluded.

Code	Category	Subcategory (first level)	Subcategory (second level)	Indication	Notes
I_ST3	Injury pattern	Severe trauma		Penetrating truncal injuries	Penetrating injuries involving the thorax, abdomen or pelvis with the potential of damaging critical internal organs or vessels.
I_ST4	Injury pattern	Severe trauma		Unstable pelvic fracture	Confirmed unstable pelvic fracture requiring surgery for stabilization or controlling hemorrhage.
I_H	Injury pattern	Hemorrhage			Injuries requiring surgery to control hemorrhage.
I_H1	Injury pattern	Hemorrhage		Clinical evidence of severe hemorrhage	Clinical observed severe active bleeding (e.g., arterial extremity bleeding requiring tourniquet application).
I_H_R	Injury pattern	Hemorrhage	Radiologic signs of major hemorrhage		
I_H_R1	Injury pattern	Hemorrhage	Radiologic signs of major hemorrhage	Ultrasonography: abdominal fluid collection	
I_H_R2	Injury pattern	Hemorrhage	Radiologic signs of major hemorrhage	CT: contrast extravasation or signs of major vessel injury	
I_H_R3	Injury pattern	Hemorrhage	Radiologic signs of major hemorrhage	CT: intra- or retroperitoneal fluid collection	

Code	Category	Subcategory (first level)	Subcategory (second level)	Indication	Notes
I_H_R4	Injury pattern	Hemorrhage	Radiologic signs of major hemorrhage	CT: severe solid organ injury	Only solid organ injuries that resulted in hemodynamic instability (as described by the authors) or required surgical intervention (as described by the authors) were included (3-5). These will primarily include grade IV or higher liver or splenic injuries. If applicable, severe pancreatic injuries may also be considered under this category.
I_CO	Injury pattern	Contamination			Injuries requiring surgery to control contamination.
I_CO1	Injury pattern	Contamination		Gustilo-Anderson grade III fractures requiring stage surgery	Includes only long bone or axial skeleton fractures, excludes rib-, hand- and foot fractures. Staged surgery is defined as a staged approach to achieve adequate fracture fixation or wound coverage. Changing of VAC dressings is not considered a surgical intervention.
I_CO2	Injury pattern	Contamination		Hollow-viscus injury or abdominal free air	
I_OT	Injury pattern	Other injury pattern			Other injuries that could not be classified under the aforementioned categories.
P	Degree of physiological insult				Abnormalities in vital parameters, physical examination, and laboratory results.

Code	Category	Subcategory (first level)	Subcategory (second level)	Indication	Notes
P_VIT	Degree of physiological insult	Abnormal vital signs			Abnormal age adjusted vital signs based on the PALS guidelines from the American Heart Association (6), or vital signs described by the authors as abnormal.
P_VIT_HD	Degree of physiological insult	Abnormal vital signs	Hemodynamic instability		Vital signs related to hemodynamic instability.
P_VIT_HD1	Degree of physiological insult	Abnormal vital signs	Hemodynamic instability	Hypotension	
P_VIT_HD2	Degree of physiological insult	Abnormal vital signs	Hemodynamic instability	Tachycardia	
P_VIT_HD3	Degree of physiological insult	Abnormal vital signs	Hemodynamic instability	Deterioration of initial hemodynamic parameters	Description of deterioration of the initial blood pressure or heart rate by the authors.
P_VIT_HD4	Degree of physiological insult	Abnormal vital signs	Hemodynamic instability	CPR on arrival	
P_VIT_HD5	Degree of physiological insult	Abnormal vital signs	Hemodynamic instability	Poor response to fluid resuscitation	Poor response to fluid resuscitation, as described by the authors.
P_VIT_HD6	Degree of physiological insult	Abnormal vital signs	Hemodynamic instability	Poor response to pelvic binding	Poor response to pelvic binding, as described by the authors.
P_VIT_ABDE	Degree of physiological insult	Abnormal vital signs	Other abnormal vital signs		Abnormal vital signs according to the ABCDE principle.
P_VIT_ABDE1	Degree of physiological insult	Abnormal vital signs	Non-hemodynamic vital signs	Threatened airway	
P_VIT_ABDE2	Degree of physiological insult	Abnormal vital signs	Non-hemodynamic vital signs	Hypoxemia	Low SpO2 or PaO2.
P_VIT_ABDE3	Degree of physiological insult	Abnormal vital signs	Non-hemodynamic vital signs	Tachypnea	
P_VIT_ABDE4	Degree of physiological insult	Abnormal vital signs	Non-hemodynamic vital signs	Deterioration of initial respiratory parameters	Description of deterioration of SpO2, PaO2 or respiratory rate by the authors.

Code	Category	Subcategory (first level)	Subcategory (second level)	Indication	Notes
P_VIT_ABDE5	Degree of physiological insult	Abnormal vital signs	Non-hemodynamic vital signs	GCS < 15 or altered consciousness	Trauma-related GCS < 15 or mentioning of altered level of consciousness or mental status.
P_VIT_ABDE6	Degree of physiological insult	Abnormal vital signs	Non-hemodynamic vital signs	Hypothermia	
P_VIT_ABDE7	Degree of physiological insult	Abnormal vital signs	Non-hemodynamic vital signs	Other aberrant vital signs	
P_LAB	Degree of physiological insult	Abnormal laboratory parameters			Abnormal laboratory findings based on reference values from the NVKC (Dutch Society of Clinical Chemistry), or description of abnormal laboratory parameters by the authors.
P_LAB_C	Degree of physiological insult	Abnormal laboratory parameters	Abnormal coagulation studies		
P_LAB_C1	Degree of physiological insult	Abnormal laboratory parameters	Abnormal coagulation studies	Low PT/aPTT/INR (static)	
P_LAB_C2	Degree of physiological insult	Abnormal laboratory parameters	Abnormal coagulation studies	Abnormal ROTEM (dynamic)	
P_LAB_C3	Degree of physiological insult	Abnormal laboratory parameters	Abnormal coagulation studies	Low fibrinogen	
P_LAB_C4	Degree of physiological insult	Abnormal laboratory parameters	Abnormal coagulation studies	Thrombocytopenia	
P_LAB_AB	Degree of physiological insult	Abnormal laboratory parameters	Acid base distubances		Disturbances in pH, lactate, BE or bicarbonate levels.
P_LAB_AB1	Degree of physiological insult	Abnormal laboratory parameters	Acid base distubances	Acidosis	pH < 7.35
P_LAB_AB2	Degree of physiological insult	Abnormal laboratory parameters	Acid base distubances	Elevated lactate	
P_LAB_AB3	Degree of physiological insult	Abnormal laboratory parameters	Acid base distubances	Low bicarbonate levels	Includes low bicarbonate, high base deficit or low base excess values.

Code	Category	Subcategory (first level)	Subcategory (second level)	Indication	Notes
P_LAB_AN		Abnormal laboratory parameters	Anemia or drop in Hb/Ht		
P_LAB_OT	Degree of physiological insult	Abnormal laboratory parameters	Other		Other laboratory values relevant to specific injury patterns (e.g., amylase, ALT/AST in cases of upper abdominal trauma).
P_PE	Degree of physiological insult	Abnormal physical exam			Findings during the physical exam that do not involve basic vital parameters (according to the PALS guidelines) and are related to the trauma.
P_PE1	Degree of physiological insult	Abnormal physical exam		Abdominal pain or tenderness	Severe abdominal pain or tenderness related to the traumatic event.
P_PE2	Degree of physiological insult	Abnormal physical exam		Abdominal distention or signs of ACS	Abdominal distention related to the traumatic event or description of the presence of abdominal compartment syndrome.
P_PE3	Degree of physiological insult	Abnormal physical exam		Signs of poor peripheral circulation	If related to the trauma, these signs may include prolonged capillary refill time, cold and clammy skin, mottled skin, and cyanosis.
P_PE4	Degree of physiological insult	Abnormal physical exam		Suspected spine injury	
P_PE5	Degree of physiological insult	Abnormal physical exam		Other abnormal signs during physical exam	
R	Resuscitation or interventions given in the ED				
R1	Resuscitation or interventions given in the ED			Intubation	

**Code**	**Category**	**Subcategory (first level)**	**Subcategory (second level)**	**Indication**	**Notes**
R2	Resuscitation or interventions given in the ED			Oxygen therapy	Only included when given to correct hypoxemia related to trauma.
R3	Resuscitation or interventions given in the ED			Crystalloid fluid resuscitation	Only included when the fluids are administered for the purpose of resuscitation.
R4	Resuscitation or interventions given in the ED			Blood transfusion	Includes resuscitation with erythrocytes, plasma or mass transfusion.
R5	Resuscitation or interventions given in the ED			TXA administration	
R6	Resuscitation or interventions given in the ED			Coagulation factor(s) or thrombocyte administration	
R7	Resuscitation or interventions given in the ED			Other resuscitation or interventions given in the ED	Specific interventions that contribute to trauma resuscitation but are not part of the general initial management (e.g., REBOA).
OT	Other indications				Indications that cannot be classified under any of the above categories (e.g., specific composite trauma scores).

APPENDIX C: Overview of included studies, excluded patients and extracted data

APPENDIX C1: Included studies and extracted data

**Author(s) (year)**	**Country**	**Study design**	**Patients (n)**	**Included (n)[a]**	**Age (y)**	**MOI (n)[b]**	**Intervention type**	**Critical appraisal[c]**	**Extracted indications (n)**	**Coded indications (n)[d]**
Atici et al. (2020) (7)	Syria	Case report	1	1	9	- Bomb explosion (1)	- DCL<br>- Complex wound management	Level: 5	- Foreign body entrance hole (1)<br>- CT: Foreign body (1)<br>- CT: Abdominal free air (1)<br>- Severe flap-like skin defect needing emergent plastic surgery (1)<br>- S_aO_2 92% (1)<br>- RR 38/min (1)<br>- Temperature 38.2 (1)<br>- Anemia (1)<br>- NaCl 0.9% 20mL/kg (1)<br>- PTS 8 (1)	- I_ST3 (1)<br>- I_ST3 (-)<br>- I_CO2 (1)<br>- I_OT (1)<br><br>- P_VIT_ABDE2 (1)<br>- P_VIT_ABDE3 (1)<br>- P_VIT_ABDE7 (1)<br>- P_LAB_AN (1)<br>- R3 (1)<br>- OT (1)
Ayub et al. (2020) (8)	USA	Case report	1	1	14	- Crush injury (1)	- DCL	Level: 5	- X-ray: Pneumoperitoneum<br>- CT: Pneumoperitoneum<br>- CT: Intraabdominal free fluid<br>- CT: Pneumomediastinum	- I_CO2 (1)<br>- I_CO2 (-)<br>- I_H_R3 (1)<br>- I_OT (1)
Buckley et al. (1996) (9)	USA	Case series	20	16	2-16	- RTA<br>- GSW<br>- Fall<br>- Crush injury<br>- Lawn-mower injury	- DCO<br>- Complex wound management	Level: 4	- Gustilo Anderson grade IIIA-C fractures needing staged surgery for adequate soft tissue coverage (16)	- I_CO1 (16)

Author(s) (year)	Country	Study design	Patients (n)	Included (n)[a]	Age (y)	MOI (n)[b]	Intervention type	Critical appraisal[c]	Extracted indications (n)	Coded indications (n)[d]
Chao et al. (2012) (10)	Hong Kong	Case series	7	4	10-16	- RTA (2)<br>- Fall (2)	- DCO	Level: 4	- ISS ≥ 15 (4)<br>- Multitrauma (3)<br>- Unstable pelvic fracture (4)<br>- Positive FAST for abdominal fluid (2)<br>- Hypotension (4)<br>- Poor response to fluid resuscitation (3)<br>- Poor response to pelvic binding (3)<br>- Crystalloid fluid administration of 30mL/kg (3)	- I_ST1 (4)<br>- I_ST2 (3)<br>- I_ST4 (4)<br>- I_H_R1 (2)<br>- P_VIT_HD1 (4)<br>- P_VIT_HD5 (3)<br>- P_VIT_HD6 (3)<br>- R3 (3)
DeCou et al. (1999) (11)	USA	Case report	1	1	5	- RTA (1)	- DCL	Level: 5	- Multitrauma: IVC laceration, renal fracture, lumbar fracture (1)<br>- CT: Contrast extravasation near the renal vessels (1)<br>- CT: Retroperitoneal hematoma (1)<br>- HR 160/min (1)<br>- Deterioration of blood pressure (1)<br>- Abdominal pain (1)	- I_ST2 (1)<br>- I_H_R2 (1)<br>- I_H_R3 (1)<br>- P_VIT_HD2 (1)<br>- P_VIT_HD3 (1)<br>- P_PE1 (1)
Demir et al. (2023) (12)	Turkey	Case series	9	4	6-11	- GSW (4)	- DCO<br>- Complex wound management	Level: 4	- Gustilo Anderson grade IIIA-C fractures needing staged surgery for adequate soft tissue coverage (4)	- I_CO1 (4)
Dumire & Morrissey (2017) (13)	USA	Case report	1	1	1	- GSW (1)	- DCL	Level: 5	- Gunshot blast to thoraco-abdominal region and left upper extremity (1)<br>- Penetrating thoraco-abdominal injury with palpable subcutaneous pellets in the abdominal region (1)<br>- Development of refractory tachycardia (1)<br>- Progressive abdominal distention (1)	- I_ST2 (1)<br>- I_ST3 (1)<br>- P_VIT_HD3 (1)<br>- P_PE2 (1)
Erdmann et al. (2000) (14)	USA	Case series	16	3	3-6	- Lawn-mower injury (3)	- DCO<br>- Complex wound management	Level: 4	- Multiple fractures: Femur, tibia and fibula (1)<br>- Gustilo Anderson grade IIIA-C fractures needing staged surgery for adequate soft tissue coverage (3)	- I_ST2 (1)<br>- I_CO1 (3)

Author(s) (year)	Country	Study design	Patients (n)	Included (n)[a]	Age (y)	MOI (n)[b]	Intervention type	Critical appraisal[c]	Extracted indications (n)	Coded indications (n)[d]
Hill et al. (2014) (15)	USA	Case series	13	2	14	- RTA (2)	- DCO	Level: 4	- Salter–Harris III and IV fractures with compartment syndrome needing staged surgery (EF → ORIF) due to severe skin swelling and blistering (2)	- I_CO1 (2)
Holcomb et al. (2005) (16)	USA	Case report	1	1	17	- RTA (1)	- DCL	Level: 5	- Mid-humeral amputation of the left arm, pulmonary contusions, and abdominal and pelvic free fluid (1)<br>- Need for tourniquet placement around the left arm (1)<br>- Ultrasound: Free fluid in the pelvis and right upper quadrant of the abdomen (1)<br>- Hypotension (1)<br>- Tachycardia (1)<br>- GCS 12 (1)<br>- Abdominal distention (1)<br>- Crystalloid fluid resuscitation (1)<br>- PRBC transfusion (1)	- I_ST2 (1)<br>- I_H1 (1)<br>- I_H_R1 (1)<br>- P_VIT_HD1 (1)<br>- P_VIT_HD2 (1)<br>- P_VIT_ABDE5 (1)<br>- P_PE2 (1)<br>- R3 (1)<br>- R4 (1)
Hu et al. (2024) (17)	China	Case report	1	1	9	- Fall (1)	- DCO	Level: 5	- Comminuted Gustilo-Anderson grade IIIB femur fracture with extrusion of a large femoral segment, requiring staged surgery for definitive fixation (1)<br>- Blood transfusion (1)	- I_CO1 (1)<br>- R4 (1)
Hurie et al. (2010) (18)	USA	Case report	1	1	9	- Fall (1)	- DCL	Level: 5	- CT: IVC disruption (1)<br>- CT: Retroperitoneal hematoma (1)<br>- SBP 80mmhg (1)<br>- Confusion (1)<br>- Fall in hematocrit (1)<br>- Abdominal tenderness (1)<br>- Crystalloid fluid resuscitation (1)	- I_H_R2 (1)<br>- I_H_R3 (1)<br>- P_VIT_HD1 (1)<br>- P_VIT_ABDE5 (1)<br>- P_LAB_AN (1)<br>- P_PE1 (1)<br>- R3 (1)

Author(s) (year)	Country	Study design	Patients (n)	Included (n)[a]	Age (y)	MOI (n)[b]	Intervention type	Critical appraisal[c]	Extracted indications (n)	Coded indications (n)[d]
Kanda et al. (2022) (19)	Japan	Case report	1	1	14	- Fall (1)	- DCL	Level: 5	- ISS 41<br>- Horseshoe kidney injury, cerebral hematoma, right pneumothorax, multiple rib fractures, thoracolumbar fractures and pelvic fracture with spinal cord injury, left femur fracture (1)<br>- CT: Contrast extravasation around the horseshoe kidney (1)<br>- CT: Retroperitoneal hematoma (1)<br>- Unmeasurable blood pressure, SBP 75mmHg after REBOA (1)<br>- HR 132/min (1)<br>- SpO2 89% (1)<br>- GCS 6 (1)<br>- Temperature 34.6 (1)<br>- Fibrinogen 139mg/dL (1)<br>- pH 7.0<br>- Lactic acid 14.1mmol/L (1)<br>- Intubation (1)<br>- Blood transfusion (1)<br>- REBOA (1)	- I_ST1 (1)<br>- I_ST2 (1)<br>- I_H_R2 (1)<br>- I_H_R3 (1)<br>- P_VIT_HD1 (1)<br>- P_VIT_HD2 (1)<br>- P_VIT_ABDE2 (1)<br>- P_VIT_ABDE5 (1)<br>- P_VIT_ABDE6 (1)<br>- P_LAB_C3 (1)<br>- P_LAB_AB1 (1)<br>- P_LAB_AB2 (1)<br>- R1 (1)<br>- R4 (1)<br>- R7 (1)

Author(s) (year)	Country	Study design	Patients (n)	Included (n)[a]	Age (y)	MOI (n)[b]	Intervention type	Critical appraisal[c]	Extracted indications (n)	Coded indications (n)[d]
Kobayashi et al. (2016) (20)	Japan	Case report	1	1	8	- RTA (1)	- DCL	Level: 5	- Liver injury in combination with trochanteric femur fracture (1)<br>- CT: Contrast extravasation hepatic artery (1)<br>- CT: Grade IV parenchymal liver disruption in a hemodynamic unstable patient (1)<br>- HR 157/min (1)<br>- Blood pressure drop from 109/71 to 77/57mmHg (1)<br>- Unstable hemodynamics despite fluid resuscitation (1)<br>- RR 30/min (1)<br>- Temperature 34.6 (1)<br>- INR 2.78 (1)<br>- Fibrinogen 34mg/dL (1)<br>- Thrombocytes 1.7 * 10^4/μL (1)<br>- pH 7.131 (1)<br>- Lactate 7.2mmol/L (1)<br>- BE -15.08mmol/L and bicarbonate 13.6mmol/L (1)<br>- Hemoglobin before resuscitation 10.8g/dL (1)<br>- Moderate transaminitis (1)<br>- Abdominal pain (1)<br>- Abdominal distention (1)<br>- Pale skin (1)<br>- 2x30mL/kg lactated Ringer's infusion (1)<br>- 1400mL PRBC transfusion (1)	- I_ST2 (1)<br>- I_H_R2 (1)<br>- I_H_R4 (1)<br>- P_VIT_HD2 (1)<br>- P_VIT_HD3 (1)<br>- P_VIT_HD5 (1)<br>- P_VIT_ABDE3 (1)<br>- P_VIT_ABDE6 (1)<br>- P_LAB_C1 (1)<br>- P_LAB_C3 (1)<br>- P_LAB_C4 (1)<br>- P_LAB_AB1 (1)<br>- P_LAB_AB2 (1)<br>- P_LAB_AB3 (1)<br>- P_LAB_AN (1)<br>- P_LAB_OT (1)<br>- P_PE1 (1)<br>- P_PE2 (1)<br>- P_PE3 (1)<br>- R3 (1)<br>- R4 (1)

Author(s) (year)	Country	Study design	Patients (n)	Included (n)[a]	Age (y)	MOI (n)[b]	Intervention type	Critical appraisal[c]	Extracted indications (n)	Coded indications (n)[d]
Luo et al. (2022) (21)	China	Case series	4	2	3, 9	- RTA (2)	- DCL	Level: 5	- Ultrasonography: Abdominal effusion (2)<br>- CT: Retroperitoneal air (2)<br>- Elevated amylase/lipase (2)<br>- Severe abdominal pain (2)	- I_H_R1 (2)<br>- I_CO2 (2)<br>- P_LAB_OT (2)<br>- P_PE1 (2)
MacKay et al. (2021) (22)	USA	Case report	1	1	17	- GSW (1)	- DCL<br>- DCT	Level: 5	- Thoracic GSW (1)<br>- CPR on arrival (1)<br>- ED thoracotomy with pericardial tamponade treatment and aortic cross clamping, followed by clamshell thoracotomy to staple a hole in the apex of the left ventricle (1)	- I_ST3 (1)<br>- P_VIT_HD4 (1)<br>- R7 (1)
Mauro et al. (2024) (23)	Brazil	Case report	1	1	14	- Stab injury (1)	- DCL	Level: 5	- Abdominal stab wound<br>- Evisceration of abdominal contents (1)<br>- Ultrasonography: FAST positive for perihepatic fluid (1)<br>- Initiation of the transfusion protocol (1)	- I_ST3 (1)<br>- I_ST3 (-)<br>- I_H_R1 (1)<br>- R4 (1)
Mayers et al. (2020) (24)	USA	Case series	6	3	-	-	- DCO<br>- Complex wound management	Level: 4	- Gustilo-Anderson IIIB tibial fractures requiring extensive VAC therapy, skin flaps and skin grafting (3)	- I_CO1 (3)
Mennen et al. (2024) (25)	Nether-lands	Case series	11	5	7-14	- RTA (4)<br>- Fall (1)	- DCO<br>- Complex wound management (n=2)	Level: 4	- ISS ≥ 15 (5)<br>- Multiple injuries in addition to pelvic fracture (5)<br>- Unstable pelvic fracture (5)<br>- Gustilo-Anderson IIIA pelvic fracture requiring staged surgery for definitive fixation (ExFix → ORIF) (4)	- I_ST1 (5)<br>- I_ST2 (5)<br>- I_ST4 (5)<br>- I_CO1 (4)

Author(s) (year)	Country	Study design	Patients (n)	Included (n)[a]	Age (y)	MOI (n)[b]	Intervention type	Critical appraisal[c]	Extracted indications (n)	Coded indications (n)[d]
Miyake et al. (2024) (26)	Japan	Case report	1	1	11	- RTA (1)	- DCL	Level: 5	- Ultrasonography: FAST positive for peri-splenic free fluid (1)<br>- CT: Contrast extravasation (1)<br>- CT: Grade V liver laceration in a hemodynamic unstable patient with massive contrast extravasation (1)<br>- Deterioration of systolic blood pressure and HR (1)<br>- RR 30/min (1)<br>- GCS 14 (1)<br>- Temperature 34.4 after transportation (1)<br>- REBOA (1)	- I_H_R1 (1)<br>- I_H_R2 (1)<br>- I_H_R4 (1)<br>- P_VIT_HD3 (1)<br>- P_VIT_ABDE3 (1)<br>- P_VIT_ABDE5 (1)<br>- P_VIT_ABDE6 (1)<br>- R7 (1)
Mooney (2012) (27)	USA	Case series	2	2	7, 13	- RTA (2)	- DCO	Level: 4	- Acetabular fracture, pubis ramus fracture, medial malleolar fracture, liver laceration and pulmonary contusion in addition to femur fracture (1)<br>- Gustilo-Anderson IIIB femoral fracture requiring staged surgery for definitive fixation (2)	- I_ST2 (1)<br>- I_CO1 (1)
Mubarak & Kanagarat-nam (2023) (28)	Sri Lanka	Case report	1	1	13	- Animal bite (1)	- DCO	Level: 5	- Severely contaminated Gustilo-Anderson IIIA tibia and fibula fracture (1)	- I_CO1 (1)

Author(s) (year)	Country	Study design	Patients (n)	Included (n)[a]	Age (y)	MOI (n)[b]	Intervention type	Critical appraisal[c]	Extracted indications (n)	Coded indications (n)[d]
Perks & Grewal (2005) (29)	USA	Case report	1	1	4	- RTA (1)	- DCL	Level: 5	- CT: abdominal injury and pneumothorax (1)<br>- CT: intra- and retroperitoneal fluid (1)<br>- HR 124/min (1)<br>- Deterioration of blood pressure and HR (1)<br>- GCS 11 (1)<br>- Abdominal distention and difficulty to ventilate (1)<br>- Intubation (1)<br>- Oxygen therapy (1)<br>- 2x20mL/kg NaCl 0.9% infusion (1)	- I_ST2 (1)<br>- I_H_R3 (1)<br>- P_VIT_HD2 (1)<br>- P_VIT_HD3 (1)<br>- P_VIT_ABDE5 (1)<br>- P_PE2 (1)<br>- R1 (1)<br>- R2 (1)<br>- R3 (1)
Polites et al. (2017) (30)	USA	Observational study	2,989	360	Mean 16	-	- DCL	Level: 3<br><br>MINORS: 9/16	Compared to the definitive laparotomy group, patients who underwent DCL had:<br>- Higher ISS: 25 vs. 18 ($p < 0.001$)<br>- Lower SBP: 104 vs. 113mmHg ($p < 0.001$)<br>- Higher HR: 112 vs. 100bpm ($p < 0.001$)<br>- Lower temperature: 34 vs. 36 degrees Celsius ($p < 0.001$)<br>- Lower GCS: 12 vs. 13 ($p < 0.001$)<br>- More transfusion: 19 vs. 11% ($p < 0.001$)	NA
Reff (1984) (31)	USA	Case series	17	6	6-12	-	- DCO<br>- Complex wound management	Level: 4	- Polytrauma (2)<br>- Long bone fractures in two distinct anatomic areas (1)<br>- Gustilo-Anderson grade III fractures of the lower extremities requiring staged surgery for adequate soft tissue coverage (6)	- I_ST2 (3)<br>- I_ST2 (-)<br>- I_CO1 (6)

Author(s) (year)	Country	Study design	Patients (n)	Included (n)[a]	Age (y)	MOI (n)[b]	Intervention type	Critical appraisal[c]	Extracted indications (n)	Coded indications (n)[d]
Rinker et al. (2004) (32)	USA	Case series	26	12	-	-	- DCO<br>- Complex wound management	Level: 4	- Gustillo-Anderson IIIB/C fractures of the tibia and fibula requiring staged surgery for adequate soft tissue coverage (12)	- I_CO1 (12)
Samms et al. (2010) (33)	Iraq	Case report	1	1	16	- GSW (1)	- DCL	Level: 5	- Sacral loss and rectal perforation (1)<br>- Pelvic GSW (1)<br>- Near complete loss of the sacrum (1)<br>- Large rectal vault deficit (1)<br>- Hypovolemic shock (1)	- I_ST2 (1)<br>- I_ST3 (1)<br>- I_ST4 (1)<br>- I_CO2 (1)<br>- P_VIT_HD1 (1)
Schwartz et al. (2018) (34)	USA	Case report	1	1	17	- GSW (1)	- DCL	Level: 5	- Multiple GSW affecting the thorax, abdomen and extremities (1)<br>- Thoraco-abdominal GSW (1)<br>- CT: Contrast extravasation in the upper abdomen (1)<br>- CT: Suspected hollow viscus injury (1)<br>- Hypotension (1)<br>- Tachycardia (1)	- I_ST2 (1)<br>- I_ST3 (1)<br>- I_H_R2 (1)<br>- I_CO2 (1)<br>- P_VIT_HD1 (1)<br>- P_VIT_HD2 (1)

Author(s) (year)	Country	Study design	Patients (n)	Included (n)[a]	Age (y)	MOI (n)[b]	Intervention type	Critical appraisal[c]	Extracted indications (n)	Coded indications (n)[d]
Soma et al. (2021) (35)	Japan	Case report	1	1	8	- RTA (1)	- DCL	Level: 5	- CT: Thyroid, grade IV liver and intestinal injury with bilateral hemopneumothorax (1)<br>- Ultrasonography: FAST fluid collection suggestive of hemorrhage (1)<br>- CT: Suggestive for intestinal injury (1)<br>- HR 140/min (1)<br>- RR 44/min (1)<br>- GCS 13, restless (1)<br>- pH 7.3 (1)<br>- Lactate 24 mmol/L (1)<br>- Bicarbonate 19.9mmol/L and BE -4.9 mmol/L (1)<br>- Hemoglobin 9.6 g/dL (1)<br>- Severe upper abdominal pain (1)<br>- Intubation (1)<br>- Oxygen therapy (1)<br>- Crystalloid fluid resuscitation (1)<br>- Blood transfusion with PRBC and plasma (1)	- I_ST2 (1)<br>- I_H_R1 (1)<br>- I_CO2 (1)<br>- P_VIT_HD2 (1)<br>- P_VIT_ABDE3 (1)<br>- P_VIT_ABDE5 (1)<br>- P_LAB_AB1 (1)<br>- P_LAB_AB2 (1)<br>- P_LAB_AB3 (1)<br>- P_LAB_AN<br>- P_PE1 (1)<br>- R1 (1)<br>- R2 (1)<br>- R3 (1)<br>- R4 (1)

Author(s) (year)	Country	Study design	Patients (n)	Included (n)[a]	Age (y)	MOI (n)[b]	Intervention type	Critical appraisal[c]	Extracted indications (n)	Coded indications (n)[d]
Stuckey et al. (2020) (36)	USA	Case report	1	1	3	- NAT (1)	- DCL	Level: 5	- Liver and IVC lacerations, and signs of brain injury (1)<br>- Ultrasonography: FAST shows large volume of intraperitoneal fluid (1)<br>- CT: IVC laceration (1)<br>- CT: hemoperitoneum (1)<br>- CT: multiple and large liver lacerations in a hemodynamic unstable patient (1)<br>- Deterioration of blood pressure (120/70 → 60/30 mmHg) and HR (120/min → 160/min) (1)<br>- No response to crystalloid fluid resuscitation (1)<br>- Deterioration of initial respiratory parameters (tachypnea and decreased S_pO_2) (1)<br>- GCS 6 (1)<br>- Hypothermia (1)<br>- Impaired pupillary light reflex on the right (1)<br>- pH 7.0 (1)<br>- Lactate 11.0 mmol/L<br>- Hb 9.5 g/dL, Ht 0.30 (1)<br>- Abdominal distention (1)<br>- Intubation (1)<br>- Crystalloid fluid resuscitation (1)<br>- Blood transfusion (1)<br>- Epinephrine administration (1)	- I_ST2 (1)<br>- I_H_R1 (1)<br>- I_H_R2 (1)<br>- I_H_R3 (1)<br>- I_H_R4 (1)<br>- P_VIT_HD3 (1)<br>- P_VIT_HD5 (1)<br>- P_VIT_ABDE4 (1)<br>- P_VIT_ABDE5 (1)<br>- P_VIT_ABDE6 (1)<br>- P_VIT_ABDE7 (1)<br>- P_LAB_AB1 (1)<br>- P_LAB_AB2 (1)<br>- P_LAB_AN (1)<br>- P_PE2 (1)<br>- R1 (1)<br>- R3 (1)<br>- R4 (1)<br>- R7 (1)

Author(s) (year)	Country	Study design	Patients (n)	Included (n)[a]	Age (y)	MOI (n)[b]	Intervention type	Critical appraisal[c]	Extracted indications (n)	Coded indications (n)[d]
Stylianos et al. (1990) (37)	USA	Case report	1	1	3	- RTA (1)	- DCL	Level: 5	- Clinical evident bleeding from abdominal drain inserted in referring hospital (1)<br>- Disruption of the left liver lobe with severe bleeding needing surgical intervention (1)<br>- SBP 60 mmHg (1)<br>- Temperature 33 degrees Celsius (1)<br>- pH 7.05 (1)<br>- Hematocrit 0.13 (1)	- I_H1 (1)<br>- I_H1 (-)<br>- P_VIT_HD1 (1)<br>- P_VIT_ABDE6 (1)<br>- P_LAB_AB1 (1)<br>- P_LAB_AN (1)
Suda & Fritsch (2023) (38)	Austria	Case report	1	1	14	- Fall (1)	- DCL	Level: 5	- CT: Injury to multiple intra-abdominal organs, pneumothorax and entero-thorax (1)<br>- CT: Rupture of multiple solid organs, including the spleen, liver, pancreatic tail, left kidney and diaphragm in a hemodynamic instable patient (1)<br>- CT: Gastric rupture (1)<br>- Shock, based on blood pressure and tachycardia (1)<br>- Deterioration of RR (1)<br>- Lactate 3.0 mmol/L (1)<br>- BE -5.5 mmol/L (1)<br>- Crystalloid fluid resuscitation (1)<br>- Tranexamic acid administration (1)<br>- Prothrombin complex administration (1)<br>- Catecholamine administration (1)	- I_ST2 (1)<br>- I_H_R4 (1)<br>- I_CO2 (1)<br>- P_VIT_HD1 (1) and P_VIT_HD2 (1)<br>- P_VIT_ABDE4 (1)<br>- P_LAB_AB2 (1)<br>- P_LAB_AB3 (1)<br>- R3 (1)<br>- R5 (1)<br>- R6 (1)<br>- R7 (1)

Author(s) (year)	Country	Study design	Patients (n)	Included (n)[a]	Age (y)	MOI (n)[b]	Intervention type	Critical appraisal[c]	Extracted indications (n)	Coded indications (n)[d]
Switzer et al. (2013) (39)	Canada	Case report	1	1	14	- RTA (1)	- DCL	Level: 5	- CT: Extensive comminuted liver laceration in a hemodynamic unstable patient (1)<br>- Blood pressure 72/32 mmHg (1)<br>- HR 120/min (1)<br>- RR 22/min (1)<br>- Hemoglobin 101g/L (1)	- I_H_R4 (1)<br><br><br>- P_VIT_HD1 (1)<br>- P_VIT_HD2 (1)<br>- P_VIT_ABDE3 (1)<br>- P_LAB_AN (1)
Villalobos et al. (2017) (40)	USA	Observational study	371	56	-	- RTA (32)<br>- Any pene-trating injury (20)<br>- GSW (7)<br>- Stab injury (2)<br>- Other (15)	- DCL	Level: 3<br>MINORS: 12/16	Compared to the definitive laparotomy group, patients who underwent DCL had:<br>- Higher ISS: 33 vs. 16 ($p < 0.0001$)<br>- Higher PATI: 29 vs. 8 ($p < 0.0001$)<br>- Lower GCS: 11.5 vs. 15 ($p < 0.0001$)<br>- Lower SBP: 92 vs. 122 mmHg ($p < 0.0001$)<br>- Lower temperature: 97.2 vs. 98 degrees Fahrenheit ($p = 0.0070$)<br>- Higher BE: 8 vs. 5 ($p < 0.0001$)<br>- More crystalloid fluid resuscitation: 2000mL vs. 700mL ($p < 0.0001$)<br>- More blood transfusion: 44.6 vs. 9.5% ($p < 0.0001$)<br>- More MTP activation: 76.8 vs. 7.3% ($p < 0.0001$)<br>- More major vascular abdominal injury: 35.7 vs. 5.7% ($p < 0.0001$)<br>- More solid organ injuries: 62.5 vs. 46.7% ($p = 0.0305$)<br>- More concomitant injuries in the form of TBI ($p = 0.0097$), extremity injury ($p = 0.0491$), pelvic fractures ($p < 0.0001$) and thoracic injuries ($p < 0.0001$)	NA

Author(s) (year)	Country	Study design	Patients (n)	Included (n)[a]	Age (y)	MOI (n)[b]	Intervention type	Critical appraisal[c]	Extracted indications (n)	Coded indications (n)[d]
Villate & Vergara (2025) (41)	Paraguay	Case report	1	1	3	- Rotation injury (1)	- DCO<br>- Complex wound management	Level: 5	- Multiple fractures: humerus, ulna and radius (1)<br>- Gustilo-Anderson IIIA fracture of the humerus with severe soft tissue injury requiring staged surgery for definitive fixation and wound coverage (1)	- I_ST2 (1)<br>- I_CO1 (1)
Vines et al. (2023) (42)	USA	Case report	1	1	10	- RTA (1)	- DCL	Level: 5	- Ultrasonography: positive FAST (1)<br>- Blood pressure 60/30 mmHg (1)<br>- HR 130/min (1)<br>- Suspected spine injury based on step off deformity and no motor/sensoric function of the lower extremities (1)<br>- MTP activation (1)	- I_H_R1 (1)<br>- P_VIT_HD1 (1)<br>- P_VIT_HD2 (1)<br>- P_PE4 (1)<br>- R4 (1)
Wang et al. (2024) (43)	China	Case series	7	5	4-14	- RTA (3)<br>- Fall (2)	- DCO	Level: 4	- ISS ≥ 15 (5)<br>- Multitrauma (5)<br>- Unstable pelvic fracture (5)<br>- Hemorrhagic shock (5)	- I_ST1 (5)<br>- I_ST2 (5)<br>- I_ST4 (5)<br>- P_VIT_HD1 (5)
Wei et al. (2024) (44)	Hong Kong	Case series	14	9	2-17	- Fall (6)<br>- RTA (3)	- DCO	Level: 4	- ISS ≥ 15 (9)<br>- Multitrauma (9)<br>- Unstable pelvic fracture (9)<br>- Ultrasonography: positive FAST (9)<br>- Hemodynamic instability / shock (9)<br>- Partial or no response to fluid resuscitation (9)	- I_ST1 (9)<br>- I_ST2 (9)<br>- I_ST4 (9)<br>- I_H_R1 (9)<br>- P_VIT_HD1 (9)<br>- P_VIT_HD5 (9)
Wiener & Abdulla (2020) (45)	Australia	Case report	1	1	13	- RTA (1)	- DCL	Level: 5	- Open book pelvic fracture (1)<br>- Ultrasonography: free fluid in the right upper quadrant (1)<br>- SBP 85 mmHg (1)<br>- HR 120/min (1)<br>- Deterioration of hemodynamic parameters (1)<br>- Unresponsive to fluid resuscitation (1)<br>- Abdominal tenderness (1)<br>- Poor peripheral circulation (1)	- I_ST4 (1)<br>- I_H_R1 (1)<br>- P_VIT_HD1 (1)<br>- P_VIT_HD2 (1)<br>- P_VIT_HD3 (1)<br>- P_VIT_HD5 (1)<br>- P_PE1 (1)<br>- P_PE3 (1)

Author(s) (year)	Country	Study design	Patients (n)	Included (n)[a]	Age (y)	MOI (n)[b]	Intervention type	Critical appraisal[c]	Extracted indications (n)	Coded indications (n)[d]
Wolf et al. (2007) (46)	USA	Case report	1	1	17	- GSW	- DCL	Level: 5	- GSW wound in the back (T3/T4) (1)<br>- Ultrasonography: positive FAST for intraperitoneal fluid (1)<br>- Hypotension (1)<br>- Tachycardia (1)<br>- Intubation (1)	- I_ST3 (1)<br>- I_H_R1 (1)<br><br>- P_VIT_HD1 (1)<br>- P_VIT_HD2 (1)<br>- R1 (1)

Abbreviations: BE, Base Excess; CT, Computer Tomography; DCL, Damage Control Laparotomy; DCO, Damage Control Orthopedics; DCT, Damage Control Thoracic surgery; EF, External Fixation; FAST, Focussed Assessment with Sonography in Trauma; GCS, Glasgow Coma Scale; GSW, Gunshot Wound; HR, Heart Rate; INR, International Normalized Ratio; ISS, Injury Severity Score; IVC, Inferior Vena Cava; MTP, Massive Transfusion Protocol; NA, Not Applicable; NAT, Non-Accidental Trauma; ORIF, Open Reduction Internal Fixation; PATI, Penetrating Abdominal Trauma Index; PRBC, Packed Red Blood Cells; PTS, Pediatric Trauma Score; REBOA, Resuscitative Balloon Occlusion of the Aorta; RR, Respiratory Rate; RTA, Road Traffic Accident; SBP, Systolic Blood Pressure; USA, United States of America; VAC, Vacuum Assisted Closure.

[a] For the case reports and case series, these are patients who meet the predefined criteria for DCS. For the observational studies, these are patients in the DCS group.
[b] If reported in the study, the number of patients per injury type is presented.
[c] For each study, the Level of Evidence is indicated according to the grading system of the Oxford Centre for Evidence-Based Medicine (47). In the case of observational studies, the Methodological Index for Non-Randomized Studies (MINORS) is also reported (48). The scoring details of the MINORS can be found in Appendix D.
[d] The codebook is provided in Appendix B.

APPENDIX C2: Excluded patients

Study	Excluded patients	Reason
Buckley et al. (1996)	1, 3, 4, 6	No operative fracture fixation (1, 4, 6)<br>No staged surgery (3, 6)
Chao et al. (2012)	B, F, G	No clear emergency surgery performed (B)<br>Age ≥ 18 (F, G)
Demir et al. (2023)	1, 2, 4, 6, 7	No staged surgery (1, 2, 4, 6, 7)
Erdmann et al. (2000)	SB, JD, CW, SD, RD, MR, BC, DG, CCo, JM, PR, JL, BP	No staged surgery (CW, SD)<br>No treatment of long bone or axial skeleton fractures (SB, JD, RD, MR, BC, DG, CCo, JM, PR, JL, BP)
Hill et al. (2014)	1, 2, 3, 4, 5, 6, 7, 10, 11, 12, 13	No staged surgery (1, 2, 3, 4, 5, 6, 7, 10, 11, 12)<br>Age ≥ 18 (13)
Luo et al. (2022)	1, 2	No emergent surgery within 24 hours of the accident (1, 2)
Mayers et al.	Patients that did not receive staged surgery (n=3)	No staged surgery (n=3)
Mennen et al. (2024)	4, 5, 7, 8, 10, 11	Unknown if staged surgery was performed within the same hospital admission (4, 5, 7, 8, 10, 11)<br>Unknown which patients receiving ORIF were operated > 24 after the incident, patients 4, 5, 7, 8 and 10 received ORIF as primary fixation method.<br>Age ≥ 18 (5).
Reff (1984)	2, 4, 5, 7, 8, 9, 11, 12, 13, 14, 17	Patients with Gustilo-Anderson grade III fractures were prepared for skin grafts or free flaps and received staged surgery. Therefore patients with no Gustilo-Anderson grade III fractures were excluded (2, 4, 5, 7, 8, 9, 11, 12, 13, 14, 17).
Rinker et al. (2004)	Patients with no Gustilo-Anderson grade IIIB/C fracture of the tibia/fibula (n=14)	Patients with open foot fractures (n=7) or pure soft-tissue avulsion injuries (n=7) were excluded, because they did not met our definition of DCS.
Wang et al. (2024)	1, 7	No emergency surgery (1)<br>No staged surgery (7)
Wei et al. (2024)	5, 10, 11, 12, 14	Patients who were not receiving the 3-in-1 method were excluded, because it was not clear if they received DCS.
The excluded patients per study are listed. Where possible, the patient is identified by a patient number, letter, or initials, shown as a subscript (e.g., 1, 2 or A, B). If this is not possible, the number of excluded patients and the reason for exclusion are indicated (n = *).		

APPENDIX D: Critical appraisal of observational studies

Methodological index for non-randomized studies (MINORS)

1. **A clearly stated aim:** the question addressed should be precise and relevant in the light of available literature

2. **Inclusion of consecutive patients**: all patients potentially fit for inclusion (satisfying the criteria for inclusion) have been included in the study during the study period (no exclusion or details about the reasons for exclusion)

3. **Prospective collection of data**: data were collected according to a protocol established before the beginning of the study

4. **Endpoints appropriate to the aim of the study**: unambiguous explanation of the criteria used to evaluate the main outcome which should be in accordance with the question addressed by the study. Also, the endpoints should be assessed on an intention-to-treat basis.

5. **Unbiased assessment of the study endpoint**: blind evaluation of objective endpoints and double-blind evaluation of subjective endpoints. Otherwise the reasons for not blinding should be stated

6. **Follow-up period appropriate to the aim of the study**: the follow-up should be sufficiently long to allow the assessment of the main endpoint and possible adverse events

7. **Loss to follow up less than 5%:** all patients should be included in the follow up. Otherwise, the proportion lost to follow up should not exceed the proportion experiencing the major endpoint

8. **Prospective calculation of the study size**: information of the size of detectable difference of interest with a calculation of 95% confidence interval, according to the expected incidence of the outcome event, and information about the level for statistical significance and estimates of power when comparing the outcomes

Additional criteria in the case of comparative study

9. **An adequate control group**: having a gold standard diagnostic test or therapeutic intervention recognized as the optimal intervention according to the available published data

10. **Contemporary groups**: control and studied group should be managed during the same time period (no historical comparison)

11. **Baseline equivalence of groups**: the groups should be similar regarding the criteria other than the studied endpoints. Absence of confounding factors that could bias the interpretation of the results

12. **Adequate statistical analyses**: whether the statistics were in accordance with the type of study with calculation of confidence intervals or relative risk

The items are scored 0 (not reported), 1 (reported but inadequate) or 2 (reported and adequate).
The global ideal score being 16 for non-comparative studies and 24 for comparative studies (48).

Critical appraisal: Polites et al., 2017 (30)

1. **A clearly stated aim:** 2 points

2. **Inclusion of consecutive patients**: 2 points

3. **Prospective collection of data**: 0 points, no research protocol available

4. **Endpoints appropriate to the aim of the study**: 1 point, it is not described how the primary outcome is determined

5. **Unbiased assessment of the study endpoint**: 0 points, blinding is not mentioned in the methods

6. **Follow-up period appropriate to the aim of the study**: 2 points

7. **Loss to follow up less than 5%:** 2 points

8. **Prospective calculation of the study size**: 0 points, the 95% Confidence Interval is not stated

Additional criteria in the case of comparative study

9. **An adequate control group**: -

10. **Contemporary groups**: -

11. **Baseline equivalence of groups**: -

12. **Adequate statistical analyses**: -

Total: 9 / 16

Critical appraisal: Villalobos et al., 2017 (40)

1. **A clearly stated aim:** 2 points

2. **Inclusion of consecutive patients**: 2 points

3. **Prospective collection of data**: 0 points, no research protocol available

4. **Endpoints appropriate to the aim of the study**: 2 points

5. **Unbiased assessment of the study endpoint**: 0 points, blinding is not mentioned in the methods

6. **Follow-up period appropriate to the aim of the study**: 2 points

7. **Loss to follow up less than 5%:** 2 points

8. **Prospective calculation of the study size**: 2 points

Additional criteria in the case of comparative study

9. **An adequate control group**: -

10. **Contemporary groups**: -

11. **Baseline equivalence of groups**: -

12. **Adequate statistical analyses**: -

Total: 12 / 16

References

1. Roberts DJ, Bobrovitz N, Zygun DA, Ball CG, Kirkpatrick AW, Faris PD, et al. Indications for use of damage control surgery and damage control interventions in civilian trauma patients: A scoping review. J Trauma Acute Care Surg. 2015;78(6):1187-96.
2. Roberts DJ, Zygun DA, Kirkpatrick AW, Ball CG, Faris PD, Bobrovitz N, et al. A protocol for a scoping and qualitative study to identify and evaluate indications for damage control surgery and damage control interventions in civilian trauma patients. BMJ Open. 2014;4(7):e005634.
3. Moore EE CT, Malangoni M, Jurkovich GJ, Champion HR. Injury Scoring Scale: A Resource for Trauma Care Professionals. The American Association for the Surgery of Trauma; n.d.
4. Coccolini F, Catena F, Moore EE, Ivatury R, Biffl W, Peitzman A, et al. WSES classification and guidelines for liver trauma. World J Emerg Surg. 2016;11:50.
5. Coccolini F, Montori G, Catena F, Kluger Y, Biffl W, Moore EE, et al. Splenic trauma: WSES classification and guidelines for adult and pediatric patients. World J Emerg Surg. 2017;12:40.
6. Topjian AA, Raymond TT, Atkins D, Chan M, Duff JP, Joyner BL, et al. Part 4: Pediatric Basic and Advanced Life Support 2020 American Heart Association Guidelines for Cardiopulmonary Resuscitation and Emergency Cardiovascular Care. Pediatrics. 2021;147(Suppl 1).
7. Atici A, Ozkan M, Celikkaya ME, Akcora B. Successful Treatment of Major Abdominal Trauma in a 9-year-old Male due to Bomb Explosion. Chirurgia (Bucharest, Romania : 1990). 2020;115(5):690-4.
8. Ayub A, Naeem B, Ahn M, Bowen-Jallow K, Tran S. Complete gastroesophageal junction avulsion after near drowning: A case report and review of literature. Int J Surg Case Rep. 2020;76:11-3.
9. Buckley SL, Smith GR, Sponseller PD, Thompson JD, Robertson WW, Jr., Griffin PP. Severe (type III) open fractures of the tibia in children. Journal of pediatric orthopedics. 1996;16(5):627-34.
10. Chao NSY, Liu CSW, Chung KLY, Tang PMY, Tai DKC, Lee KY, et al. Retroperitoneal pelvic packing for haemodynamically unstable pelvic fractures in children and adolescents: A level-one trauma-centre experience. Journal of Pediatric Surgery. 2012;47(12):2244-50.
11. DeCou JM, Abrams RS, Gauderer MWL. Seat-belt transection of the pararenal vena cava in a 5-year-old child: Survival with caval ligation. Journal of Pediatric Surgery. 1999;34(7):1074-6.
12. Demir T, Kilinccioglu NK, Dogramaci Y. Treatment of Gustilo-Anderson type III open fractures with segmental long bone loss secondary to gunshot and war ınjurıes in paediatric patients with bone segment transport by the limb reconstructıon system (LRS). Injury. 2023;54(4):1138-43.
13. Dumire R, Morrissey S. Damage Control Vascular Surgery for the General Surgeon. Am Surg. 2017;83(1):e6-7.
14. Erdmann D, Lee B, Roberts CD, Levin LS. Management of lawnmower injuries to the lower extremity in children and adolescents. Ann Plast Surg. 2000;45(6):595-600.
15. Hill BW, Rizkala AR, Li M. Clinical and functional outcomes after operative management of Salter-Harris III and IV fractures of the proximal tibial epiphysis. Journal of pediatric orthopedics Part B. 2014;23(5):411-8.
16. Holcomb JB, Hoots K, Moore FA. Treatment of an acquired coagulopathy with recombinant activated factor VII in a damage-control patient. Mil Med. 2005;170(4):287-90.
17. Hu X, Tan Q, Zhu G, Liu K. Successful reimplantation of extruded bone segment in lower limb open fractures: case report and literature review. Front Pediatr. 2024;12:1333575.
18. Hurie J, Ehrlich P, Castle V, Eliason JL. Isolated infrarenal caval disruption secondary to minimal blunt trauma. Ann Vasc Surg. 2011;25(1):132 e13-5.
19. Kanda T, Ito K, Tsunoyama T, Nagao T, Morimura N. Ao - Ito K, https://orcid.org O. Case of horseshoe kidney injury treated with simultaneous damage control operation and endovascular therapy in a hybrid emergency system (HERS). Trauma Surgery and Acute Care Open. 2022;7(1):e000885.
20. Kobayashi T, Kubota M, Arai Y, Ohyama T, Yokota N, Miura K, et al. Staged laparotomies based on the damage control principle to treat hemodynamically unstable grade IV blunt hepatic injury in an eight-year-old girl. Surgical case reports. 2016;2(1):134.
21. Luo Y, He X, Geng L, Ouyang R, Xu Y, Liang Y, et al. Diagnosis and treatment of traumatic duodenal rupture in children. BMC gastroenterology. 2022;22(1):61.

22. MacKay EJ, Niu NT, Cannon JW, Kaplan LJ, Pascual JL. Prolonged mechanical rib separation is a key element to prevent thoracic compartment syndrome in penetrating chest trauma: A case report. Trauma Case Reports. 2021;34:100498.
23. Mauro DM, Miyazawa B, Nascimento SJ, Alves TS, Scorpione JGN. Superior mesenteric vein injury in penetrating abdominal trauma: Case report and a literature review. Trauma Case Rep. 2024;52:101045.
24. Mayers A, Dunleavy ML, Chau MM, Hennrikus W. The Vacuum-Assisted Closure Device Increases Value in the Treatment of Gustilo Grade IIIb Open Tibia Fractures in Children. Cureus. 2020;12(9):e10194.
25. Mennen AHM, Van Lieshout EMM, Bisoen PA, Bloemers FW, Geerlings AE, Koole D, et al. Long-term musculoskeletal function after Open Pelvic ring fractures in Children (OPEC); a multicentre, retrospective case series with follow-up measurement. Trauma Case Rep. 2024;52:101050.
26. Miyake Y, Okishio Y, Shibata N, Kawashima S, Nasu T, Ueda K. Survival of a hemodynamically unstable pediatric liver trauma patient with aortic balloon occlusion catheter during air transport: A case report. Acute Med Surg. 2024;11(1):e955.
27. Mooney JF. The use of 'damage control orthopedics' techniques in children with segmental open femur fractures. J Pediatr Orthop B. 2012;21(5):400-3.
28. Mubarak FS, Kanagaratnam K. Placing Locking Compression Plates as an External Fixator in Wild Animal (Crocodile) Bite Victim: A Case Report. Cureus. 2023;15(10):e47511.
29. Perks DH, Grewal H. Abdominal compartment syndrome in the pediatric patient with blunt trauma. J Trauma Nurs. 2005;12(2):50-4.
30. Polites SF, Habermann EB, Glasgow AE, Zielinski MD. Damage control laparotomy for abdominal trauma in children. Pediatric Surgery International. 2017;33(5):587-92.
31. Reff RB. The use of external fixation devices in the management of severe lower-extremity trauma and pelvic injuries in children. Clinical orthopaedics and related research. 1984(188):21-33.
32. Rinker B, Valerio IL, Stewart DH, Pu LLQ, Vasconez HC. Microvascular free flap reconstruction in pediatric lower extremity trauma: A 10-Year review. Plastic and Reconstructive Surgery. 2005;115(6):1618-24.
33. Samms S, Wittich A, Graf K. Extraordinary military medical care of a pediatric patient with penetrating pelvic trauma: a case report. Mil Med. 2010;175(11):926-8.
34. Schwartz J, Madden NJ, Butts CA, Slotman GJ, Budeir MH. The Problem of Exposure for Damage Control in an Adolescent with Multiple Abdominal Gunshot Wounds Six Months after Surgery for a Previous Abdominal Gunshot. Am Surg. 2018;84(8):e314-e6.
35. Soma J, Ishii D, Miyagi H, Ishii S, Motoki K, Kawabata H, et al. Damage control surgery for grade IV blunt hepatic injury with multiple organ damage in a child: a case report. Surgical case reports. 2021;7(1):269.
36. Stuckey ME, Abdul Ghani MO, Greeno A, Lovvorn HN, Danko M.E. Ao - Stuckey ME, https://orcid.org O. Non-accidental trauma causing inferior vena cava and liver injuries. Journal of Pediatric Surgery Case Reports. 2020;62:101649.
37. Stylianos S, Jacir NN, Hoffman MA, Harris BH. Pediatric blunt liver injury and coagulopathy managed with packs and a silo: Case report. Journal of Trauma. 1990;30(11):1409-10.
38. Suda AJ, Fritsch G. Traumatic pancreas, kidney, liver, spleen, gastric and diaphragma rupture with enterothorax after blunt trauma caused by falling in an adolescent: a case report. Arch Orthop Trauma Surg. 2023;143(8):5015-23.
39. Switzer NJ, Bigam DL, Dicken B. Case report: Management of pediatric blunt abdominal trauma following an ATV accident leading to liver hilum injury. Journal of Pediatric Surgery Case Reports. 2013;1(5):102-5.
40. Villalobos MA, Hazelton JP, Choron RL, Capano-Wehrle L, Hunter K, Gaughan JP, et al. Caring for critically injured children: An analysis of 56 pediatric damage control laparotomies. The journal of trauma and acute care surgery. 2017;82(5):901-9.
41. Medina Villate MM, Navarro Vergara AD. Floating Elbow in Children as a Serious Domestic Accident: A Case Report. Cureus. 2025;17(1):e77107.
42. Vines K, Ward M, Butts C, Capasso T, Bright A, Lee YL, et al. Aortic Injury From High-Speed Deceleration Against a Lap Belt in a 10-year-Old. Am Surg. 2023;89(8):3554-6.

43. Wang Y, Du X, Tomaszewski R, Journeau P, Mayr J. Operative management of sacroiliac joint dislocation in children with unstable pelvic fractures - A STROBE-compliant investigation. J Orthop. 2024;52:6-11.
44. Wei AYZ, King Him TC, Bong LK. Three-in-one protocol for the management of hemodynamically unstable paediatric pelvic fracture – a level one trauma centre 15 year review. Journal of Orthopaedics, Trauma and Rehabilitation. 2024;31(2):223-32.
45. Wiener J, Abdulla M. Ao - Wiener J, https://orcid.org O. Damage control laparotomy in a paediatric trauma patient in a regional hospital. International Journal of Surgery Case Reports. 2020;75:169-71.
46. Wolf JH, Miller G, Ashinoff R, Dave J, Lefleur RS, Frangos SG, et al. Pancreaticoureteral fistula following penetrating abdominal trauma. Journal of the Pancreas. 2007;8(5):613-6.
47. Howick J, Chalmers I, Glasziou P, Greenhalgh T, Heneghan C, Liberati A, Moschetti I, Phillips B, Thornton H, Goddard O, Hodgkinson M. The Oxford 2011 Levels of Evidence: Oxford Center for Evidence-Based Medicine; 2011 [Available from: https://www.cebm.ox.ac.uk/resources/levels-of-evidence/ocebm-levels-of-evidence.
48. Slim K, Nini E, Forestier D, Kwiatkowski F, Panis Y, Chipponi J. Methodological index for non-randomized studies (minors): development and validation of a new instrument. ANZ J Surg. 2003;73(9):712-6.
